# Development of a real-time PCR (qPCR) method for the identification of the invasive paddle crab *Charybdis japonica* (*Crustacea*, *Portunidae*)

**DOI:** 10.7717/peerj.15522

**Published:** 2023-06-12

**Authors:** Tiffany JS Simpson, Claire M. Wellington, Sherralee S. Lukehurst, Roger Huerlimann, Heather Veilleux, Michael Snow, Joana Dias, Justin I. McDonald

**Affiliations:** 1 Conservation and Fisheries Directorate, Ascension Island Government, Georgetown, Ascension Island, South Atlantic, United Kingdom; 2 Trace and Environmental DNA (TrEnD) Laboratory, Curtin University of Technology, Perth, Western Australia, Australia; 3 Sustainability and Biosecurity, Department of Primary Industries and Regional Development (DPIRD), Perth, Western Australia, Australia; 4 School of Biological Sciences, The University of Western Australia, Perth, Western Australia, Australia; 5 College of Science and Engineering, James Cook University, Townsville, Queensland, Australia; 6 Ecometrix Incorporated, Mississauga, Ontario, Canada; 7 Genotyping Australia, Perth, Western Australia, Australia; 8 School of Biological, Environmental and Earth Sciences, University of Southern Mississippi, Hattiesburg, Mississippi, United States of America

**Keywords:** Invasive marine species, Real-time PCR, Environmental DNA, Western Australia, CO1

## Abstract

Crabs can be transported beyond their native range *via* anthropogenic-mediated means such as aquarium trade, live seafood trade and shipping. Once introduced into new locations, they can establish persisting populations and become invasive, often leading to negative impacts on the recipient environment and native species. Molecular techniques are increasingly being used as complementary tools in biosecurity surveillance and monitoring plans for invasive species. Molecular tools can be particularly useful for early detection, rapid identification and discrimination of closely related species, including when diagnostic morphological characters are absent or challenging, such as early life stages, or when only part of the animal is available. In this study, we developed a species-specific qPCR assay, which targets the cytochrome c oxidase subunit 1 (CO1) region of the Asian paddle crab *Charybdis japonica*. In Australia, as well as many parts of the world, this species is considered invasive and routine biosecurity surveillance is conducted to reduce the risk of establishment. Through rigorous testing of tissue from target and non-target species we demonstrate that this assay is sensitive enough to detect as little as two copies per reaction and does not cross amplify with other closely related species. Field samples and environmental samples spiked with *C. japonica* DNA in high and low concentrations indicate that this assay is also a promising tool for detecting trace amounts of *C. japonica* eDNA in complex substrates, making it a useful complementary tool in marine biosecurity assessments.

## Introduction

The Asian paddle crab, *Charybdis japonica* (Milne-Edwards, 1861) is widely distributed in its native region from China and Taiwan to Japan, Thailand, Malaysia (Wee & Ng, 1995) and Korea (Sung-Hoi & Yong-Rock, 1998) and has been recently described in Bangladesh (Ahmed, Afrin & Barua, 2021). In many parts of the world, it is regarded as an invasive pest, with the most significant incursion having occurred in New Zealand (Smith et al., 2003). It was originally introduced to Auckland in 2000 (Webber, 2001; Smith et al., 2003) and subsequently spread along the coast of the North Island, with detections in new harbours or estuaries hundreds of kilometers apart every 2 to 3 years. Commercial fishermen have reported that population abundances have constantly increased in these areas, with no signs of stabilisation or decrease (Hilliam & Tuck, 2022). This incursion has caused displacement of native species such as the New Zealand paddle crab *Ovalipes catharus*, as well as burrowing urchins and bivalves (Gust & Inglis, 2006; Fowler, Gerner & Sewell, 2011; Fowler & McLay, 2013; Fowler & McLay, 2013; Hilliam & Tuck, 2022). *C. japonica* exhibits many characteristics common to invasive pests including generalist predatory feeding strategies (Jiang et al., 1998), aggressive behavior (Fowler & McLay, 2013), high reproductive output and the ability to produce multiple broods each year (Wang et al., 1996).

In its native range, *C. japonica* inhabits intertidal and sublittoral habitats. Intertidal species are easily spread by vessel pathways as they can be picked up as juveniles in ballast water, or as adults on hulls (Hewitt, Gollasch & Minchin, 2009). *C. japonica* larvae also have wide environmental tolerances (Fowler, Gerner & Sewell, 2011) and are therefore likely to survive transport in or on vessels and be physiologically able to survive in the recipient environment. The tendency for pre-moult and moulting crabs to seek out shelter may also predispose this species to transport in the crevices of ocean-going ships (Dodgshun & Coutts, 2003).

Due to the invasive potential of this species, prevention and early detection are the best management strategies to avoid unwanted introductions. They are also likely to be transported during early life stages, which can be difficult to detect and discriminate visually (Smith et al., 2003). Identification of *Charybdis* species using morphology is difficult, as some key characteristics are very similar or overlap between species (Smith et al., 2003). Therefore, molecular methods may often be better suited to the early detection and rapid, accurate identification of *C. japonica*. Real-time PCR (qPCR) has been increasingly adopted by biosecurity agencies for the detection of target invasive species around the world. The mitochondrial gene CO1 is often used for designing PCR assays as it is present in high copy numbers in the cells of most eukaryotes and it is highly conserved across species. Short processing time combined with high specificity and sensitivity provides a reliable and efficient method of detecting invasive species, including from complex environmental samples such as water and sediment (Bott et al., 2010; Dias et al., 2013; Smith et al., 2012; Loh et al., 2014; Simpson et al., 2016). qPCR assays have been developed for other crab species including *Rhithropanopeus harrisi* (Forsström & Vasemägi, 2016) and *Carcinus maenas* (Roux et al., 2020; Danziger & Frederich, 2022) for the application of biosecurity surveillance but there have been no previously published assays for the detection of *C. japonica*.

There is a high risk of *C. japonica* populations becoming established in Australia, due to the high level of vessel traffic from potential source locations in Asia (Cope et al., 2016) and suitable environmental conditions for the survival and growth of introduced larvae or adult crabs (Marine Pest Sectoral Committee, 2022). Individuals of *C. japonica* have been reported in both South Australia and Western Australia (Hourston, McDonald & Hewitt, 2015; Wiltshire et al., 2020; NIMPIS, 2022). If populations are established, this species has the potential to displace native Western Australian crab species, such as *Portunus armatus* (Lai, Ng & Davie, 2010), a highly important recreational and commercial species (Johnston et al., 2011). For these reasons, *C. japonica* is identified on the Western Australia Prevention List for Introduced Marine Pests (2016) and regular monitoring for this, and other invasive marine species, is conducted by the Department of Primary Industries and Regional Development (DPIRD) in Western Australia (WA).

While no viable population of *C. japonica* has been detected in Western Australia, one specimen was confirmed in the Peel Harvey estuary in 2010 and three more were discovered in the Swan River estuary in 2012 following a delimiting survey and public awareness campaign (Hourston, McDonald & Hewitt, 2015). In 2018, another adult specimen was collected from the Swan River estuary, leading to a thorough survey to determine whether a population had established. Crab trapping surveillance has been carried out annually since then, with no other *C. japonica* specimens detected (C. Wellington personal communication, 2022). These surveys revealed that *C. japonica* can be commonly confused with many of the native crabs found in these estuaries, particularly native *Charybdis* species. The specimens ultimately identified as *C. japonica* were killed, broken and or cooked, making identification by taxonomic means challenging (Hourston, McDonald & Hewitt, 2015).

These factors instigated the need for a molecular method to complement the current biosecurity monitoring regime. The aim of this study was to design a targeted qPCR assay that could be used for the confirmation of identity of *C. japonica* from tissue samples and potentially be used as a screening tool for environmental DNA (eDNA) detection of early life stages or trace amounts of *C. japonica* in environmental samples including seawater, sediment or settlement plate biofouling.

## Materials and Methods

### Primer and probe design

The CO1 gene was targeted for the development of the *C. japonica* qPCR assay due to an appropriate level of variation within this gene region and availability of CO1 sequences for *Charybdis* species in GenBank (www.ncbi.nlm.nih.gov/genbank/). Sequences of the currently known *C. japonica* haplotypes were aligned with sequences of 12 other similar *Charybdis* species all previously reported on GenBank (www.ncbi.nlm.nih.gov/genbank/). Accession numbers of all sequences used are listed in Table S1. An alignment of all sequences was performed in Geneious (version R10); duplicates and sequences that were too short or not in the target region were removed. Tree Builder was used to build a consensus tree which was used to identify mislabeled sequences for removal. Alignments were imported into AlleleID (PREMIER Biosoft) and the taxa specific/cross species TaqMan design algorithm was run.

In order to help guarantee the specificity of the method, assay primers and probe were tested *in silico* using the similarity-based Basic Local Alignment Search Tool (Nucleotide BLAST) to check the GenBank (National Center for Biotechnology Information, NCBI) database for any similar sequences and non-specific amplification. Primer BLAST (https://www.ncbi.nlm.nih.gov/tools/primer-blast/) was also conducted to test the specificity of the primers against a *C. japonica* sequence (GenBank accession number MN184685.1) from a taxonomically vouchered specimen within the DPIRD reference collection. Results are available in Document S1.

### Specificity

#### Sample collection and DNA barcoding

**Figure 1:**
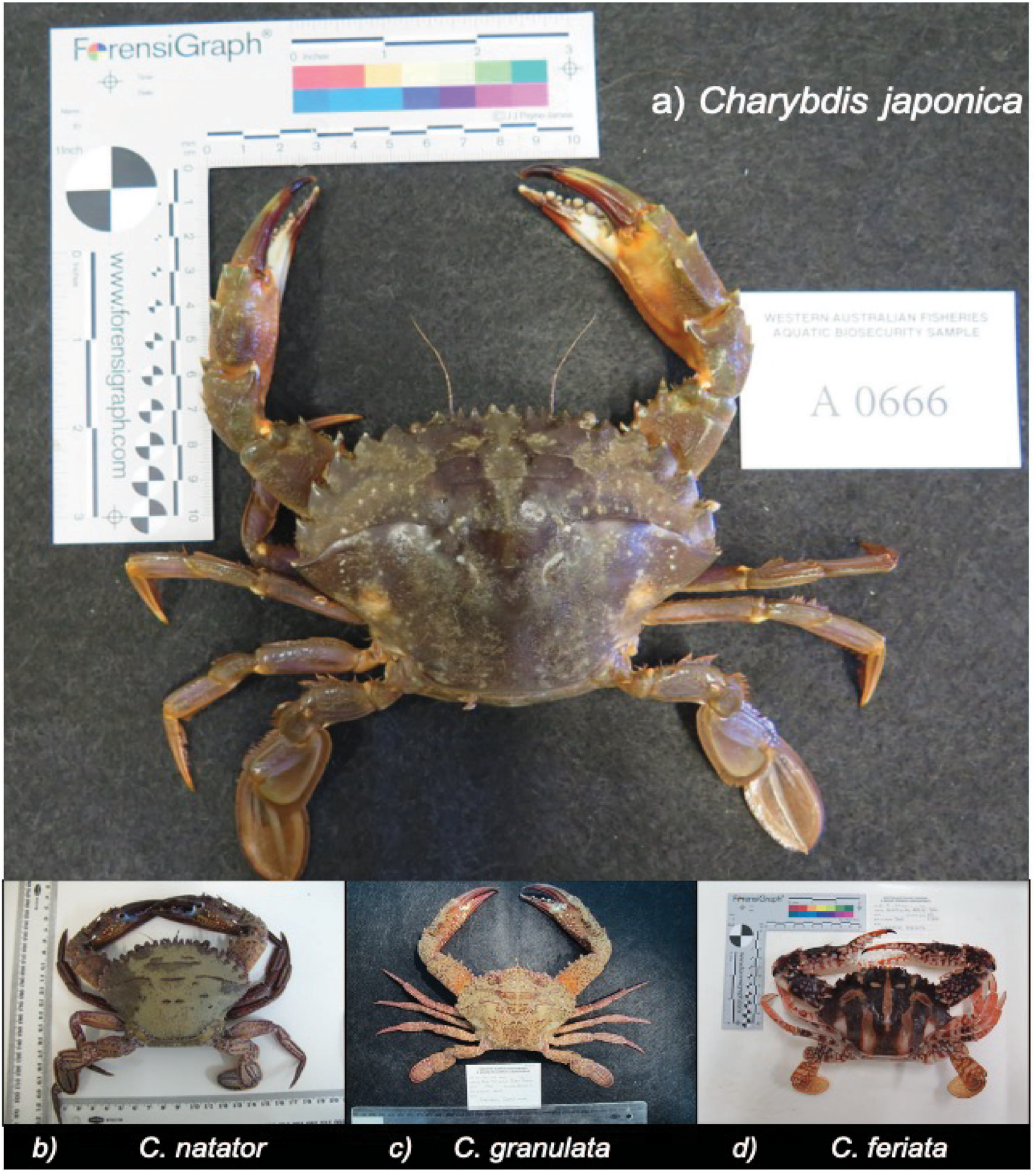
Photographs of target and often misidentified crab species (provided by DPIRD). (A) Target species *Charybdis japonica* as well as commonly mis-identified crabs (B) *Charybdis natator*, (C) *Charybdis granulata* and (D) *Charybdis feriata*. Image credit: Matthew Hewitt.

Specimens of *C. japonica* (Fig. 1), commonly misidentified swimmer crabs, invasive crab species, as well as other non-target species were obtained to test for assay specificity (Table 1). These specimens were taxonomically identified by experts and made available to the DPIRD molecular laboratory following a *C. japonica* delimiting survey conducted during 2012–2013 (Hourston, McDonald & Hewitt, 2015). DNA barcoding of each specimen was performed to confirm species identity. From each crab, DNA was extracted from ~20 mg leg tissue using a Favorgen FavorPrep Tissue Genomic DNA Extraction Mini Kit (FavorGen BioTech Corp, Taiwan), following the manufacturer’s instructions. All DNA samples were stored at −20 °C until further use.

**Table 1:** Details of Portunid and non-target taxa used in this study to test *in situ* for primer specificity and sensitivity.

Phylum	Family	Species	GenBank accession number
*Arthropoda*	*Portunidae*	*Charybdis japonica*	MN184685
		*Carcinus maenas*	MN184686
		*Charybdis acutifrons*	MN184687
		*Charybdis anisodon*	MN184688
		*Charybdis annulata*	MN184689
		*Charybdis feriata*	MN184690
		*Charybdis granulata*	MN184691
		*Charybdis hellerii*	MN184692
		*Charybdis natator*	MN184693
		*Ovalipes australiensis*	MN184694
		*Portunus armatus*	MN184695
		*Portunus sanguinolentus*	MN184696
		*Scylla serrata*	MN184697
		*Thalamita crenata*	MN184698
		*Thalamita danae*	MN184699
		*Thalamita prymna*	MN184700
		*Thalamita sima*	MN184701
	*Varundiae*	*Hemigrapsus sanguineus*	MN184702
	*Grapsidae*	*Pachygrapsus fakaravensis*	MN184703
	*Panopeidae*	*Rhithropanopeus harrisi*	MN184704
*Mollusca*	*Mytilidae*	*Perna viridis*	MN184705
		*Arcuatula senhousia*	MN184706
	*Ostreidae*	*Crassostrea gigas*	MN184707
*Annelida*	*Sabellidae*	*Sabella spallanzanii*	MN184708
*Echinodermata*	*Asteriidae*	*Asterias amurensis*	MN184709
*Chordata*	*Didemnidae*	*Didemnum perlucidum*	MN184710
	*Oxudercidae*	*Tridentiger trigoncephalus*	MN184711

To confirm specimen identity of species in Table 1, PCR amplification of the barcode CO1 gene region was performed using the LCO1490/HCO2198 primers developed by Folmer et al. (1994). PCR reactions were conducted in 25 µL containing 2 µL DNA (~20 ng), 50 µM of dNTPs (Fisher Biotec, Wembley, WA, Australia), 2.5 mM MgCl_2_ (Fisher Biotec, Wembley, WA, Australia), 1x reaction Buffer (Fisher Biotec, Wembley, WA, Australia), 10 nM of each primer, 0.04U/µL Taq DNA polymerase (Fisher Biotec, Wembley, WA, Australia) and PCR-grade water (Fisher Biotec, Wembley, WA, Australia). PCR conditions consisted of an initial incubation at 94 °C for 1 min, followed by five cycles of 94 °C for 40 s, 45 °C for 40 s and 72 °C for 60 s; 35 cycles of 94 °C for 40 s, 51 °C for 40 s, 72 °C for 60 s; and a final extension step of 72 °C for 5 min. PCR reactions were conducted in an Applied Biosystems (ABI) 2720 thermal cycler. PCR products were separated by electrophoresis using 1.5% agarose (Fisher Biotec, Wembley, WA, Australia) gels stained with GelRed (Biotium, Fisher Biotec, Wembley, WA, Australia) alongside a 100-base pair (bp) molecular weight marker (Axygen Biosciences, Union City, CA, USA) and visualised under UV light.

Bi-directional sequencing of PCR products was performed using the Sanger sequencing service provided by the Australian Genome Research Facility (AGRF, Perth). Consensus sequences were generated using the Sequencher® 5.0 sequence analysis software (Gene Codes Corporation, Ann Arbor, MI USA). Sequences were aligned, analysed and trimmed in BioEdit 7.1.3.0 (Hall, 1999). Individual species identifications were confirmed by similarity-based searches on the Barcode of Life Database (BOLD, Ratnasingham & Hebert, 2007) and the NCBI BLAST database (Altschul et al., 1990).

### Sensitivity

#### Synthetic oligonucleotide standards and limits of detection

Synthetic DNA (sDNA), to be used as a positive control, was designed to include an area 20–50 bp up and downstream of the furthest primer at each end. A stretch of 6 bp was flipped to facilitate identification of putative sDNA contamination of samples. Synthetic oligonucleotides were ordered as gBlocks DNA fragments from Integrated DNA technologies and resuspended according to manufacturer’s instructions, resulting in a starting concentration of 10 ng/µL. Efficiencies of the primers and probe, *i.e*., Efficiency (%) = [10(−1/slope)]−1 × 100], were assessed using a standard curve based on 12 replicate amplification reactions. This was conducted on a 10-fold dilution series of synthetic oligonucleotide (IDT) with a starting concentration of 10^−4^ ng/µL. The sensitivity of the assay was evaluated by calculating the limit of detection (LOD) and the limit of quantification (LOQ). Target species-specific LOD and LOQ values were calculated using the LOD/LOQ calculator script published by Klymus et al. (2019) in R (R version 4.2.1; R Core Team, 2022). Repeatability of the assay was determined by using six technical replicates of the synthetic standard at concentration of 10^−4^ ng/µL in five separate PCR runs done by three independent operators over multiple days.

#### Environmental sampling and PCR screening of eDNA

Field samples (seawater and plankton) were collected from three sites in the Swan River region: Blackwall Reach (32.019665°S 115.784231°E), Mosman Bay (32.013189°S 115.777137°E) and Perth waters (32.970211°S 115.869337°E). These sites were chosen based on the location of previously caught specimens of *C. japonica* and form ongoing surveillance sites. Sampling was conducted over 3 days in March 2019, with water samples (*n* = 3) and phytoplankton tows (*n* = 2) collected at each site (Table 2). Water samples (1 L per replicate) were collected using a 3.5 L Wilco Van Dorn sampler at a 1 m depth below the surface. For phytoplankton, a plankton net with a 20 μm mesh and 300 mm diameter was hauled over a 100 m transect and concentrated into 120 ml in a sterile container. All samples were kept on ice for approximately 2 h while in the field and were filtered on the day of collection onto 47 mm polyethersulfone filters with a 0.2 µm pore size (Pall Life Sciences; New York, NY, USA) using a Sentino peristaltic pump (Pall Life Sciences). Filters were immediately stored at −20 °C until DNA extraction. Environmental DNA (eDNA) was extracted from filters using the DNeasy PowerWater kit (QIAGEN) following the manufacturer’s instructions. An extraction control was included, and all samples were eluted in 100 µL elution buffer. All eDNA samples were screened with the *C. japonica* assay.

**Table 2:** eDNA samples (water and plankton) collected from three sites in the lower Swan River (WA) in 2019 and tested using qPCR assay for the detection of *Charybdis japonica*. All samples were negative.

Date	Sample type	Sample code	Site	qPCR result	Latitude	Longitude
18/03/2019	eDNA water sample	A0158	Blackwall Reach	Negative	−32.0166	115.7851
18/03/2019	eDNA water sample	A0157	Blackwall Reach	Negative	−32.0180	115.7848
18/03/2019	eDNA water sample	A0159	Blackwall Reach	Negative	−32.0194	115.7842
18/03/2019	Plankton tow	A0160	Blackwall Reach	Negative	−32.0183	115.7851
18/03/2019	Plankton tow	A0161	Blackwall Reach	Negative	−32.0160	115.7844
19/03/2019	eDNA water sample	A0162	Mosman Bay	Negative	−32.0075	115.7725
19/03/2019	Plankton tow	A0165	Mosman Bay	Negative	−32.0075	115.7729
19/03/2019	eDNA water sample	A0163	Mosman Bay	Negative	−32.0085	115.7731
19/03/2019	Plankton tow	A0166	Mosman Bay	Negative	−32.0096	115.7730
19/03/2019	eDNA water sample	A0164	Mosman Bay	Negative	−32.0100	115.7745
27/03/2019	eDNA water sample	A0168	B52	Negative	−31.9787	115.8359
27/03/2019	Plankton tow	A0169	B52	Negative	−31.9778	115.8367
27/03/2019	eDNA water sample	A0171	B52	Negative	−31.9787	115.8353
27/03/2019	Plankton tow	A0170	B52	Negative	−31.9793	115.8359
27/03/2019	eDNA water sample	A0172	B52	Negative	−31.9790	115.8363

Assays were conducted in a final volume of 10 µL containing 2 µL of DNA template, 1x TaqMan Fast Advanced master mix (Applied Biosystems), 900 nM of each primer and 250 nM of the probe. Assays were performed on an ABI Step One Plus qPCR system using a cycling profile of 50 °C for 2 min (UNG activation) and 95 °C for 10 min (DNA polymerase activation) followed by 40 cycles of 95 °C for 15 s (denaturation) and 60 °C for 1 min (annealing/extension). Reactions were conducted in triplicate, and all experiments included a negative qPCR control (no template DNA added) and a positive control of confirmed *C. japonica* DNA and synthetic oligonucleotide (IDT). To test for inhibition of eDNA samples, sDNA (10^−6^ ng/µL) was mixed with either neat or diluted eDNA (−910 ng/µL).

#### Spiking experiment

To demonstrate the efficacy of the assay using samples isolated from a range of substrates, two blind spiking experiments were conducted. In the first experiment, DNA extracted from *C. japonica* muscle tissue using the method described above was spiked at a ratio of 1:1,000 into previously extracted eDNA samples. A total of 34 eDNA samples were used, extracted from multiple substrates including sediment (*n* = 10), seawater (*n* = 10) and homogenized settlement plate biomass (*n* = 14) from across five locations representing very different assemblages: Broome, Garden Island and Esperance (Western Australia), Darwin (Northern Territory, Australia), and Bulgaria. Only 10 of these samples were randomly spiked with *C. japonica* DNA. All eDNA samples had been screened through 18S metabarcoding analysis to ensure that the target was not present in the sample prior to spiking (detailed methodology described in Simpson et al. (2018)).

In the second spiking experiment, *C. japonica* muscle tissue was added to fresh biological samples from marine sediment and homogenized settlement plate biomass collected from Darwin. Tissue was added to one sediment and one settlement plate sample in a ‘high’ concentration of 0.1 g tissue in 10 g substrate (0.01% w/w) as well as two sediment and two settlement plate samples with a ‘low’ concentration of 0.01 g tissue in 10 g substrate (0.001% w/w). Sample sizes were extremely low due to limited availability of tissue. Two sediment and two settlement plate samples were purposely not spiked to provide negative controls. Each sample was then homogenized and sub-sampled for extraction. Settlement plate samples were extracted using Qiagen DNeasy Blood and Tissue kits with an increased amount of starting material (100–200 mg), Buffer ATL (1,260 µL) and Pro-K (140 µL) but otherwise following manufacturer’s instructions. The sediment samples were extracted using Qiagen DNeasy Powersoil extraction kit, following manufacturer’s instructions. Extraction controls were included using each kit protocol. Samples were eluted in 100 µL DNase/RNase free H_2_O and stored at 4 °C until qPCR testing. Combinations of spiked and non-spiked samples were randomly transferred into a 96 well plate and qPCR test was conducted. qPCRs were carried out in 20 µL reactions containing 5 µL of DNA template, 1x Taqpath ProAmp Master Mix (Life Technologies Australia Pty Ltd, Welshpool, WA, Australia), 900 nM of each primer and 250 nM of the probe. Assays were performed on a Quant Studio 3 qPCR system (Life Technologies Australia Pty Ltd, Welshpool, WA, Australia). The cycling profile consisted of an initial denaturation step of 10 min at 95 °C, followed by 40 cycles of 15 s at 95 °C and 60 s at 60 °C. Reactions were conducted in triplicate and included a negative template control and a synthetic oligonucleotide standard curve as a positive control.

## Results

### qPCR assay development, optimization and testing

The initial alignment comprised 274 sequences, which were reduced to 221 sequences after length trimming to 695 bp, removal of short sequences and/or sequences outside the gene region, and removal of redundant sequences (Table S1). The consensus tree built from the alignment identified further sequences that needed to be removed. One sequence of *C. japonica* (KM987387) strongly clustered with sequences of *C. annulata* and one sequence of *C. variegata* (EU284142) strongly clustered with *C. japonica*. It is highly likely that these sequences were misidentified when uploaded to NCBI and were therefore removed from the assay design process. Five further *C. japonica* sequences (HM180497, HM180498, HM180499, HM180500, HM180501) clustered away from the rest, and were also removed from the assay design. The assay was designed in AlleleID and modified in Geneious (Table 3); the assay amplified a 139 bp fragment.

**Table 3:** Sequences of primers and TaqMan-MGB probe. Primer and probe name, sequence, attributed dye (probe), melting temperature (Tm, °C), GC content (%) and length (bp).

Name	Sequence 5′-3′	Label	Tm C	%GC	Length (bp)
Cjap XL F	TTAATATACGGTCATTTGGTATGAGTATAGATC	_	59.5	30.3	33
Cjap XL R	AAGTTTCGGTCTGTTAATAATATAGTAATAGCT	_	59.4	27.3	33
Cjap X probe	TATTACTGCCATTCTTCTAC	FAM	69.0	50	20

Results from the *in-silico* evaluation of primer and probe specificity indicated that the designed qPCR assay was specific for *C. japonica*. To further validate the primers and probes, 20 distinct crab species were successfully barcoded, including the target species, *C. japonica* and seven congener *Charybdis* species that have similar characteristics and are found living in similar environmental conditions (Hourston, McDonald & Hewitt, 2015). *Portunus armatus* and *Scylla serrata* were also barcoded as commercially important species, and four Thalamita species were included as commonly abundant species in Western Australia. Four invasive crab species, *Carcinus maenas*, *Hemigrapsus sanguineus*, *Pachygrapsus fakaravensis* and *Rhithropanopeus harrisi*, listed on the Western Australian Prevention list for Introduced Marine Pests (2016) were also included. These species, as well as seven non-target taxa, were screened with the qPCR assay. *C. japonica* samples generated positive cycle threshold (Ct) values (Ct range 16–22) and there was no cross amplification with any of the other species tested (Table 1).

**Figure 2:**
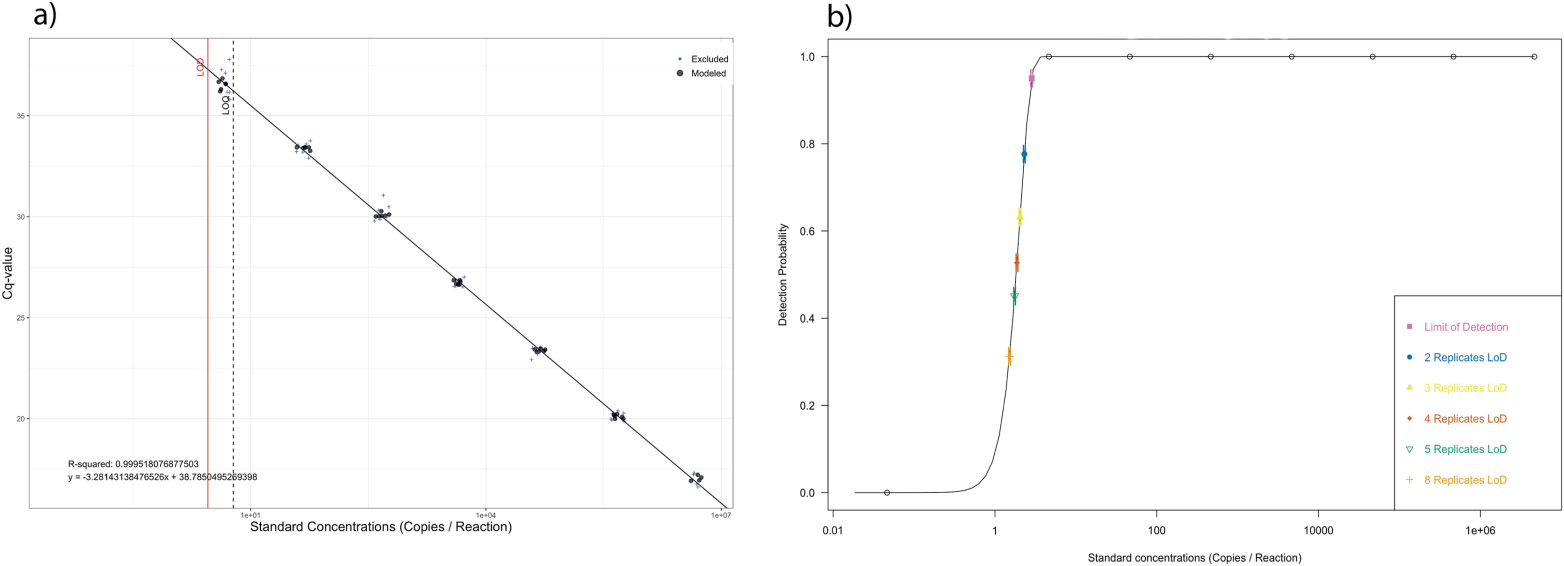
Standard curve and LOD plots. (A) Standard Curve plot with black circles representing the replicates of standards that were included in the linear regression calculations, the blue + indicated those reactions that were excluded from the linear regression calculations. The LOD of the assay is indicated by the solid line and the LOQ by the dotted line. (B) LOD plot indicating detection probability of each standard tested and coloured points represent the effective LOD for multiple replicate analysis as determined by a two parameter Weibell type 2 function.

To obtain accurate and comparable results, qPCR efficiency should be as close to 100% (slope of −3.33) as possible (Pfaffl, 2004; Valasek & Repa, 2005). Standard curves revealed slope values of −3.28 (101.78% efficiency) for *C. japonica* synthetic oligonucleotide (Fig. 2A, Table S2). There was a high correlation between cycle number and dilution factor, R^2^ = 0.9995. The modelled LOD and LOQ were calculated as 2.84 and six copies per a reaction respectively (Fig. 2A). Multiple replicate testing analysis shows that with more replicates the LOD of the assay is lower; the effective LOD using three qPCR replicates for each sample was 2.04 copies per reaction (Fig. 2B). For a measure of repeatability, coefficient of variation of Cts was calculated to be 5.7%.

### qPCR screening of eDNA samples

Comprehensive trapping using opera house and commercial crab traps carried out in 2019 did not detect any *C. japonica* specimens in the Swan River. All eDNA samples collected from the Swan River region were also negative for *C. japonica*, indicating that it is unlikely there is a viable population of *C. japonica* crabs present in the river, and there was no cross amplification with other crab species present in the samples. When field samples were spiked with 10^−6^ ng/µL sDNA to test for inhibition, all neat and diluted eDNA samples had positive amplifications with Ct’s around 26–27, which was the same Ct range produced for the 10^−6^ ng/µL sDNA alone, thus indicating a lack of inhibition by the eDNA matrix.

### qPCR screening of spiked eDNA samples

**Figure 3:**
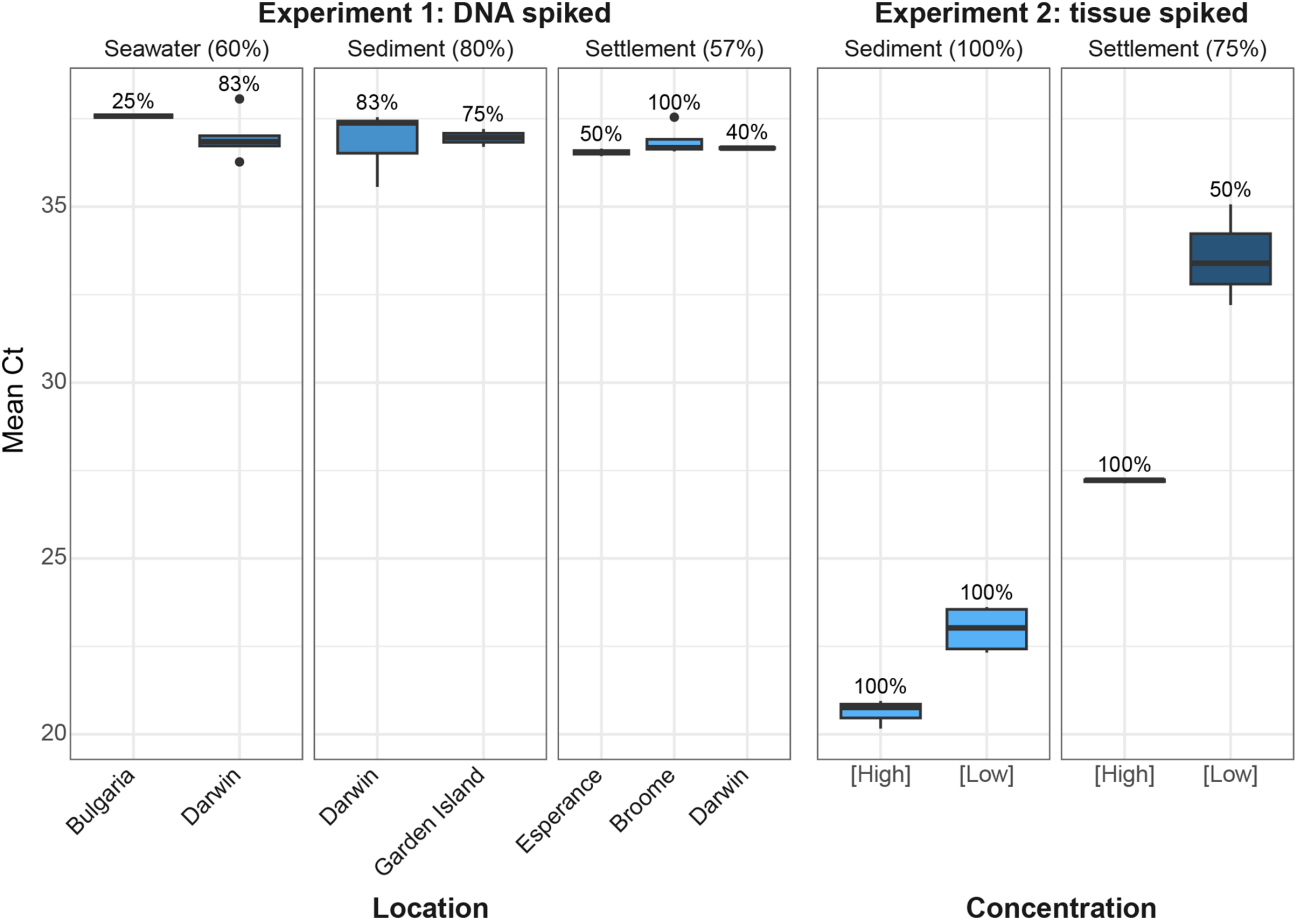
Mean qPCR Ct values for Experiment 1 and Experiment 2. Experiment 1 eDNA samples were collected from sediment (*n* = 10), settlement plates (n = 14), and seawater (*n* = 10) from Broome, Garden Island and Esperance (Western Australia), Broome (Northern Territory, Australia) and Bulgaria, and were spiked with *C. japonica* DNA. Experiment 2 samples from sediment (*n* = 3) and settlement plates (*n* = 3) were spiked with high and low concentrations (0.01% w/w and 0.001%, respectively) of *C. japonica* muscle tissue. The percentage of positive detections are indicated in brackets above each box, with lighter shades in boxes representing a higher proportion of passing replicates. For each box, the thick horizontal black line, lower hinge and upper hinge represent the 50^th^, 25^th^ and 75^th^ percentiles, respectively. The upper and lower whiskers are the maximum and minimum values, respectively, and dots are outliers.

From the first experiment, a sample was considered to be positive if any of the three technical replicates resulted in a positive amplification with a normal curve that crossed the cycle threshold. Six of the 10 spiked water samples (60%), eight of the 14 spiked settlement plate samples (57%) and eight of the 10 spiked sediment samples (80%) resulted in positive amplification, with Ct values ranging between 35–38 (Fig. 3). Positive detections were Sanger sequenced and all were a 100% match to the target *C. japonica*. There were no positive detections in un-spiked samples, indicating that there was no cross amplification with other crab species.

From the second spiking experiment, two of the three settlement plate samples detected *C. japonica* in all three replicates (one high concentration, one low concentration) with Ct values ranging between 25–35. One of the low concentration samples did not amplify. This may have been a result of poor homogenization of the original sample or inhibition. All replicates of the three sediment samples resulted in strong amplification with Ct values ranging from 20–25 (Fig. 3).

## Discussion

Our results demonstrate that the qPCR assay developed in this study is specific for the identification of the invasive crab *Charybdis japonica*. Using qPCR, we were able to discriminate this species from others that commonly occur in Western Australia and are often misidentified using morphologic techniques. This assay was determined to be discriminatory in identifying *C. japonica* using muscle tissue samples. This study also demonstrated the diagnostic performance of the assay in being sensitive enough to detect this species in complex environmental samples including seawater, settlement plate biomass and sediment.

Information derived from molecular analyses often surpasses conventional approaches in taxonomic resolution and sensitivity and there is great capacity for complementing marine biosecurity programs (Zaiko et al., 2018). In a criteria-based assessment of potential tools for deriving biosecurity relevant information, target-specific tools like qPCR scored very highly in overall performance for marine biosecurity applications (Zaiko et al., 2018). However, field validation of molecular assays in a low-density target population can be challenging. A range of factors should always be considered such as seasonal fluctuations, potential false positives (of yet unrecognized native species), appropriate field methodology, replication, and acknowledgement of potentially low quantities of target eDNA. Previous studies have also indicated that some species such as crabs have lower rates of shedding DNA into the environment, which can limit the ability to detect their presence (Forsström & Vasemägi, 2016; Roux et al., 2020) Even with these challenges, molecular techniques add valuable complementary information alongside conventional survey techniques (Wood et al., 2019).

International vessels (sea chests and ballast water), recreational fishing vessels and aquaculture are assumed to be the likely sources of introduction of *C. japonica* into New Zealand (Gust & Inglis, 2006; Hilliam & Tuck, 2022), Western Australia (Hourston, McDonald & Hewitt, 2015) and South Australia (Wiltshire et al., 2020), and trade statistics indicate an increase in vessel traffic originating in the native range of *C. japonica* (Cope et al., 2016). Sea water temperatures are also increasing globally, which may facilitate further spread of this species into new regions that may have previously been inhospitable for larval survival and development (Fowler, Gerner & Sewell, 2011; Hewitt, Hourston & McDonald, 2018). The increasing vectors of movement and likelihood of establishment of this species combined with its aggressive and competitive characteristics (Fowler & McLay, 2013) provide motivation for routine surveillance and monitoring regimes to protect against further incursions of *C. japonica*.

For these reasons, this assay has been integrated into the marine biosecurity monitoring practices of the Western Australian Department of Primary Industries and Regional Development (WA DPIRD), not only as a diagnostic tool to confirm the identity of suspect adult specimens, but also as a screening tool for *C. japonica* eDNA, including larvae, in routine plankton tows and biofouling on settlement plates. Environmental sampling for marine biosecurity surveillance using water, plankton and biofouling have been tested with this assay in Western Australia since 2021. Annual surveillance for *C. japonica* in the Swan River WA using eDNA sampling was undertaken in 2021 and 2022 at three sites, in conjunction with targeted crab trapping surveys within the estuary. There were no detections of *C. japonica* from the eDNA sampling (plankton tows and eDNA water samples) nor any live animals captured in the crab traps surveys. Despite ongoing public awareness campaigns and numerous public reports from recreational fishers, there have been no detections of *C. japonica* in Western Australia since 2018 (C. Wellington, personal communication. 2022). Not only does this suggest that there have been no new incursions of this species in WA waters, despite increased temperatures and La Nina events (Hewitt, Hourston & McDonald, 2018), but it also demonstrates there has been no cross amplification in using this assay for mixed environmental samples.

The first occurrence of *C. japonica* in the Swan River estuary resulted in a public awareness campaign and a delimiting survey that included over 20,000 trap hours and countless hours of identifying specimens misidentified by members of the public (Hourston, McDonald & Hewitt, 2015). Despite the massive sampling effort, one of the limitations of the approach at that time was that it targeted only adults. With the addition of the molecular technique presented in this study, routine water and plankton sampling at the likely introduction sites have expanded the probability of detecting incursions of *C. japonica* at earlier life stages (*e.g*., larvae and juveniles) and tracking distributions with more targeted sampling effort. Early detection through rapid and effective identification of this invasive marine species is imperative for confirming its presence and distribution in the environment, as well as initiating a timely response to an incursion (Marine Pest Sectoral Committee, 2022). eDNA monitoring in combination with traditional sampling methods are becoming more routine in determining distribution limits of invasive species and informing management decisions (Bylemans et al., 2016; Furlan & Gleeson, 2016). Frequent surveys and commercial fishing efforts may be sufficient for early detection of adult invasive species but in areas of low frequency surveying and limited recreational fishing, molecular methods may increase the likelihood of detection even when the DNA prevalence is low. In South Australia, retrospective testing of plankton samples using the *C. japonica* qPCR assay detected the presence of this species in two samples from Lipson Reach. These samples collected in 2017 pre-date the subsequent reporting and capture of two adult specimens of *C. japonica* by commercial and recreational fishers in the Gulf St Vincent, SA (Wiltshire et al., 2020).

As with any molecular method, care must be taken in the interpretation of results. Further research is required to understand the detection limits of this method on complex samples, as only 60–80% of the spiked samples resulted in positive detections. This may have been due to a number of factors including the amount and the quality of the tissue available for spiking, the homogeneity of the spiked samples, the potential inhibition, the quality of the complex sample into which the *C. japonica* tissue was spiked and the small sample size. A negative result cannot conclusively prove complete absence of an invasive species in a particular location. However, this qPCR assay can provide rapid confirmation of species identification, as well as provide additional lines of evidence for the early detection of *C. japonica*, when applied to environmental DNA sampling. A positive result may also be used to guide more extensive surveys beyond routine surveillance. Incorporation of this assay as a complementary tool into the larger biosecurity surveillance program can be beneficial for the rapid response and early detection of invasive species.

Targeted eDNA assays require extensive validation through *in silico*, *in vitro* and *in situ* testing in order to provide meaningful application and interpretation (Thalinger et al., 2021). However, despite the limitations of field validation in an area where the density of the target species is inherently low, we believe this assay has been put through rigorous testing and can be used with a high degree of confidence.

## Conclusion

This study has developed a qPCR assay for detection of *C. japonica* DNA. Through rigorous testing, it has been demonstrated as a reliable method for discriminating this species from closely related crab species and is sensitive in detecting *C. japonica* in complex environmental samples. Using this molecular technique in combination with traditional sampling adds confidence in identifying specimens and increases the ability to detect *C. japonica* at early life stages or in scarce abundance. This method has already been adopted into the routine sampling regime for biosecurity surveillance in Western Australia and will continue to be a powerful tool for detecting incursions and preventing establishment and spread of a harmful invasive species.

## Supplemental Information

10.7717/peerj.15522/supp-1 Supplemental Information 1 Detailed list of Genbank accession numbers of target (bold) and exclusion species of the *C. japonica* assay.

10.7717/peerj.15522/supp-2 Supplemental Information 2 LOD and standard curve results.

10.7717/peerj.15522/supp-3 Supplemental Information 3 Results from Primer Blast on *Charybdis japonica* assay.

## Additional Information and Declarations

### DNA Deposition

The following information was supplied regarding the deposition of DNA sequences: The sequences are available at GenBank (Table S1): MN184685–MN184711.

### Data Availability

The following information was supplied regarding data availability: The qPCR results, including LOD and standard curve results, are available in the Supplemental File.
