# Supplementary material for: Development of a real-time PCR (qPCR) method for the identification of the invasive paddle crab Charybdis japonica (Crustacea, Portunidae)
Source: PeerJ. 2023 Jun 12;11:e15522. doi: 10.7717/peerj.15522 (PMC10269569; doi:10.7717/peerj.15522)
Supplement: Supplemental Information 3 [file peerj-11-15522-s003.pdf]

An official website of the United States government

Here's how you know

The .gov means it's official.
Federal government websites often end in .gov or .mil. Before sharing sensitive information, make sure you're on a federal government site.

The site is secure.
The **https://** ensures that you are connecting to the official website and that any information you provide is encrypted and transmitted securely.

Skip to main page content

Access keys NCBI Homepage MyNCBI Homepage Main Content Main Navigation

Log in

Primer-BLAST » JOB ID:i4FUOHMgfohZtmSzadNAgRPIUbM-20quPw

Primer-BLAST Results
Help

•

Input PCR template
MN184685.1 Charybdis japonica isolate Biosec714 cytochrome oxidase subunit I (COI) gene, partial cds; mitochondrial

Range
1 - 652

Specificity of primers
Primers may **not** be specific to the input PCR template as targets were found in selected database:Nucleotide collection (nt) ...help on specific primers

Other reports
Search Summary

Graphical view of primer pairs +−

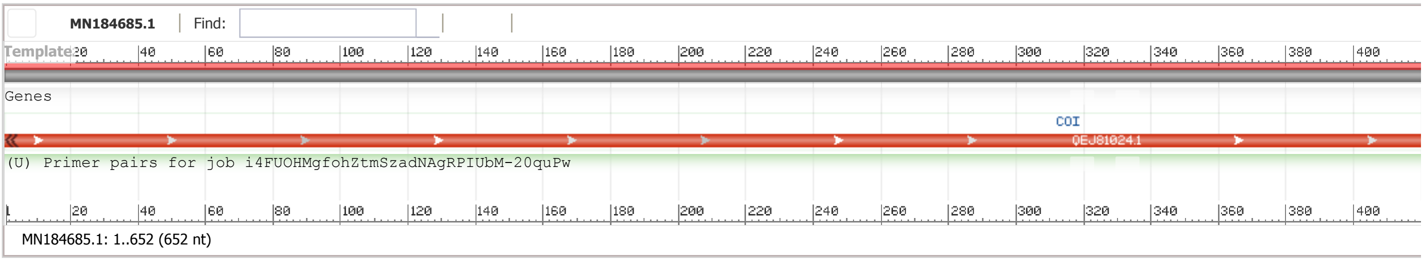

Detailed primer reports

You can re-search for specific primers by accepting some of the unintended targets, check the box(es) next to the ones you accept and try again to re-search for specific primers Submit

Help

Primer pair 1

	Sequence (5'->3')	Template strand	Length	Start	Stop	Tm	GC%	Self complementarity	Self 3' complementarity
**Forward primer**	TTAATATACGGTCATTTGGTATGAGTATAGATC	Plus	33	457	489	59.49	30.30	5.00	4.00
**Reverse primer**	AAGTTTCGGTCTGTTAATAATATAGTAATAGCT	Minus	33	595	563	59.53	27.27	8.00	6.00
**Internal oligo**		Plus							
**Product length**	139								
**Product Tm**									
**Product Tm - min(OLIGO Tm)**									
**Exon junction**									
**Total intron size**									

Products on intended targets

>MN184685.1 Charybdis japonica isolate Biosec714 cytochrome oxidase subunit I (COI) gene, partial cds; mitochondrial

```
product length = 139
Forward primer  1    TTAATATACGGTCATTTGGTATGAGTATAGATC  33
Template        457  .................................  489

Reverse primer  1    AAGTTTCGGTCTGTTAATAATATAGTAATAGCT  33
Template        595  .................................  563
```

Products on allowed targets

Products on allowed transcript variants

Products on potentially unintended templates

>OL876973.1 Charybdis japonica voucher NSMK-MS-0024816 cytochrome c oxidase subunit I (COX1) gene, partial cds; mitochondrial

```
product length = 139
Forward primer  1    TTAATATACGGTCATTTGGTATGAGTATAGATC  33
Template        483  .................................  515

Reverse primer  1    AAGTTTCGGTCTGTTAATAATATAGTAATAGCT  33
Template        621  .................................  589
```

>KX060214.1 Charybdis japonica isolate XDNA - 506 442 cytochrome oxidase subunit 1 (COI) gene, partial cds; mitochondrial

```
product length = 139
Forward primer  1    TTAATATACGGTCATTTGGTATGAGTATAGATC  33
Template        470  .................................  502

Reverse primer  1    AAGTTTCGGTCTGTTAATAATATAGTAATAGCT  33
Template        608  .................................  576
```

>KT365716.1 Charybdis japonica isolate E173 cytochrome oxidase subunit I (COI) gene, partial cds; mitochondrial

```
product length = 139
Forward primer  1    TTAATATACGGTCATTTGGTATGAGTATAGATC  33
Template        458  .................................  490

Reverse primer  1    AAGTTTCGGTCTGTTAATAATATAGTAATAGCT  33
Template        596  .................................  564
```

>KP976229.1 Gaetice depressus voucher ihb201306408 cytochrome oxidase subunit 1 (COI) gene, partial cds; mitochondrial

```
product length = 139
Forward primer  1    TTAATATACGGTCATTTGGTATGAGTATAGATC  33
Template        467  .................................  499

Reverse primer  1    AAGTTTCGGTCTGTTAATAATATAGTAATAGCT  33
Template        605  .................................  573
```

>KP976228.1 Gaetice depressus voucher ihb201306410 cytochrome oxidase subunit 1 (COI) gene, partial cds; mitochondrial

```
product length = 139
Forward primer  1    TTAATATACGGTCATTTGGTATGAGTATAGATC  33
Template        467  .................................  499

Reverse primer  1    AAGTTTCGGTCTGTTAATAATATAGTAATAGCT  33
Template        605  .................................  573
```

>KP976227.1 Gaetice depressus voucher ihb201306411 cytochrome oxidase subunit 1 (COI) gene, partial cds; mitochondrial

```
product length = 139
Forward primer  1    TTAATATACGGTCATTTGGTATGAGTATAGATC  33
Template        467  .................................  499

Reverse primer  1    AAGTTTCGGTCTGTTAATAATATAGTAATAGCT  33
Template        605  .................................  573
```

>KP976226.1 Gaetice depressus voucher ihb201306412 cytochrome oxidase subunit 1 (COI) gene, partial cds; mitochondrial

```
product length = 139
Forward primer  1    TTAATATACGGTCATTTGGTATGAGTATAGATC  33
Template        467  .................................  499

Reverse primer  1    AAGTTTCGGTCTGTTAATAATATAGTAATAGCT  33
Template        605  .................................  573
```

>KP976225.1 Gaetice depressus voucher ihb201306316 cytochrome oxidase subunit 1 (COI) gene, partial cds; mitochondrial

```
product length = 139
Forward primer  1    TTAATATACGGTCATTTGGTATGAGTATAGATC  33
Template        467  .................................  499

Reverse primer  1    AAGTTTCGGTCTGTTAATAATATAGTAATAGCT  33
Template        605  .................................  573
```

>KP976224.1 Gaetice depressus voucher ihb201306317 cytochrome oxidase subunit 1 (COI) gene, partial cds; mitochondrial

```
product length = 139
Forward primer  1    TTAATATACGGTCATTTGGTATGAGTATAGATC  33
Template        467  .................................  499

Reverse primer  1    AAGTTTCGGTCTGTTAATAATATAGTAATAGCT  33
Template        605  .................................  573
```

>KP976223.1 Gaetice depressus voucher ihb201306321 cytochrome oxidase subunit 1 (COI) gene, partial cds; mitochondrial

```
product length = 139
Forward primer  1    TTAATATACGGTCATTTGGTATGAGTATAGATC  33
Template        467  .................................  499

Reverse primer  1    AAGTTTCGGTCTGTTAATAATATAGTAATAGCT  33
Template        605  .................................  573
```

>KP976222.1 Gaetice depressus voucher ihb201306323 cytochrome oxidase subunit 1 (COI) gene, partial cds; mitochondrial

```
product length = 139
Forward primer  1    TTAATATACGGTCATTTGGTATGAGTATAGATC  33
Template        467  .................................  499

Reverse primer  1    AAGTTTCGGTCTGTTAATAATATAGTAATAGCT  33
Template        605  .................................  573
```

>KP976221.1 Gaetice depressus voucher ihb201306401 cytochrome oxidase subunit 1 (COI) gene, partial cds; mitochondrial

```
product length = 139
Forward primer  1    TTAATATACGGTCATTTGGTATGAGTATAGATC  33
Template        467  .................................  499

Reverse primer  1    AAGTTTCGGTCTGTTAATAATATAGTAATAGCT  33
Template        605  .................................  573
```

>KP976220.1 Gaetice depressus voucher ihb201306405 cytochrome oxidase subunit 1 (COI) gene, partial cds; mitochondrial

```
product length = 139
Forward primer  1    TTAATATACGGTCATTTGGTATGAGTATAGATC  33
Template        467  .................................  499

Reverse primer  1    AAGTTTCGGTCTGTTAATAATATAGTAATAGCT  33
Template        605  .................................  573
```

>KP976219.1 Gaetice depressus voucher ihb201306406 cytochrome oxidase subunit 1 (COI) gene, partial cds; mitochondrial

```
product length = 139
Forward primer  1    TTAATATACGGTCATTTGGTATGAGTATAGATC  33
Template        467  .................................  499
```

```
Reverse primer  1    AAGTTTCGGTCTGTTAATAATATAGTAATAGCT  33
Template        605  .................................  573
```

>KP976218.1 Gaetice depressus voucher ihb201306407 cytochrome oxidase subunit 1 (COI) gene, partial cds; mitochondrial

```
product length = 139
Forward primer  1    TTAATATACGGTCATTTGGTATGAGTATAGATC  33
Template        467  .................................  499

Reverse primer  1    AAGTTTCGGTCTGTTAATAATATAGTAATAGCT  33
Template        605  .................................  573
```

>KM377986.1 Charybdis japonica haplotype 14 cytochrome oxidase subunit I (COI) gene, partial cds; mitochondrial

```
product length = 139
Forward primer  1    TTAATATACGGTCATTTGGTATGAGTATAGATC  33
Template        459  .................................  491

Reverse primer  1    AAGTTTCGGTCTGTTAATAATATAGTAATAGCT  33
Template        597  .................................  565
```

>KM377985.1 Charybdis japonica haplotype 13 cytochrome oxidase subunit I (COI) gene, partial cds; mitochondrial

```
product length = 139
Forward primer  1    TTAATATACGGTCATTTGGTATGAGTATAGATC  33
Template        459  .................................  491

Reverse primer  1    AAGTTTCGGTCTGTTAATAATATAGTAATAGCT  33
Template        597  .................................  565
```

>KM377984.1 Charybdis japonica haplotype 12 cytochrome oxidase subunit I (COI) gene, partial cds; mitochondrial

```
product length = 139
Forward primer  1    TTAATATACGGTCATTTGGTATGAGTATAGATC  33
Template        459  .................................  491

Reverse primer  1    AAGTTTCGGTCTGTTAATAATATAGTAATAGCT  33
Template        597  .................................  565
```

>KM377983.1 Charybdis japonica haplotype 11 cytochrome oxidase subunit I (COI) gene, partial cds; mitochondrial

```
product length = 139
Forward primer  1    TTAATATACGGTCATTTGGTATGAGTATAGATC  33
Template        459  .................................  491

Reverse primer  1    AAGTTTCGGTCTGTTAATAATATAGTAATAGCT  33
Template        597  .................................  565
```

>KM377982.1 Charybdis japonica haplotype 10 cytochrome oxidase subunit I (COI) gene, partial cds; mitochondrial

```
product length = 139
Forward primer  1    TTAATATACGGTCATTTGGTATGAGTATAGATC  33
Template        459  .................................  491

Reverse primer  1    AAGTTTCGGTCTGTTAATAATATAGTAATAGCT  33
Template        597  .................................  565
```

>KM377978.1 Charybdis japonica haplotype 6 cytochrome oxidase subunit I (COI) gene, partial cds; mitochondrial

```
product length = 139
Forward primer  1    TTAATATACGGTCATTTGGTATGAGTATAGATC  33
Template        459  .................................  491

Reverse primer  1    AAGTTTCGGTCTGTTAATAATATAGTAATAGCT  33
Template        597  .................................  565
```

>KM377977.1 Charybdis japonica haplotype 5 cytochrome oxidase subunit I (COI) gene, partial cds; mitochondrial

```
product length = 139
Forward primer  1    TTAATATACGGTCATTTGGTATGAGTATAGATC  33
Template        459  .................................  491

Reverse primer  1    AAGTTTCGGTCTGTTAATAATATAGTAATAGCT  33
Template        597  .................................  565
```

>KM377976.1 Charybdis japonica haplotype 4 cytochrome oxidase subunit I (COI) gene, partial cds; mitochondrial

```
product length = 139
Forward primer  1    TTAATATACGGTCATTTGGTATGAGTATAGATC  33
Template        459  .................................  491

Reverse primer  1    AAGTTTCGGTCTGTTAATAATATAGTAATAGCT  33
Template        597  .................................  565
```

>KM377975.1 Charybdis japonica haplotype 3 cytochrome oxidase subunit I (COI) gene, partial cds; mitochondrial

```
product length = 139
Forward primer  1    TTAATATACGGTCATTTGGTATGAGTATAGATC  33
Template        459  .................................  491

Reverse primer  1    AAGTTTCGGTCTGTTAATAATATAGTAATAGCT  33
Template        597  .................................  565
```

>MW446892.1 Charybdis japonica mitochondrion, complete genome

```
product length = 139
Forward primer  1    TTAATATACGGTCATTTGGTATGAGTATAGATC  33
Template        500  .................................  532

Reverse primer  1    AAGTTTCGGTCTGTTAATAATATAGTAATAGCT  33
Template        638  .................................  606
```

>HQ848917.1 Uncultured Jiaozhou Bay zooplankton clone JC76 cytochrome oxidase subunit 1 (COX1) gene, partial cds; mitochondrial

```
product length = 139
Forward primer  1    TTAATATACGGTCATTTGGTATGAGTATAGATC  33
Template        264  .................................  296

Reverse primer  1    AAGTTTCGGTCTGTTAATAATATAGTAATAGCT  33
Template        402  .................................  370
```

>HM180588.1 Gaetice depressus voucher NSMK:MS-000662 cytochrome oxidase subunit I (COI) gene, partial cds; mitochondrial

```
product length = 139
Forward primer  1    TTAATATACGGTCATTTGGTATGAGTATAGATC  33
Template        484  .................................  516

Reverse primer  1    AAGTTTCGGTCTGTTAATAATATAGTAATAGCT  33
Template        622  .................................  590
```

>HM237603.1 Charybdis japonica haplotype hap7 cytochrome oxidase subunit I-like (COI) gene, partial sequence; mitochondrial

```
product length = 139
Forward primer  1    TTAATATACGGTCATTTGGTATGAGTATAGATC  33
Template        458  .................................  490

Reverse primer  1    AAGTTTCGGTCTGTTAATAATATAGTAATAGCT  33
Template        596  .................................  564
```

>HM237602.1 Charybdis japonica haplotype hap6 cytochrome oxidase subunit I (COI) gene, partial cds; mitochondrial

```
product length = 139
Forward primer  1    TTAATATACGGTCATTTGGTATGAGTATAGATC  33
Template        459  .................................  491

Reverse primer  1    AAGTTTCGGTCTGTTAATAATATAGTAATAGCT  33
Template        597  .................................  565
```

>HM237601.1 Charybdis japonica haplotype hap5 cytochrome oxidase subunit I (COI) gene, partial cds; mitochondrial

```
product length = 139
Forward primer  1    TTAATATACGGTCATTTGGTATGAGTATAGATC  33
Template        459  .................................  491

Reverse primer  1    AAGTTTCGGTCTGTTAATAATATAGTAATAGCT  33
Template        597  .................................  565
```

>HM237600.1 Charybdis japonica haplotype hap4 cytochrome oxidase subunit I (COI) gene, partial cds; mitochondrial

```
product length = 139
Forward primer  1    TTAATATACGGTCATTTGGTATGAGTATAGATC  33
Template        459  .................................  491

Reverse primer  1    AAGTTTCGGTCTGTTAATAATATAGTAATAGCT  33
Template        597  .................................  565
```

>HM237599.1 Charybdis japonica haplotype hap3 cytochrome oxidase subunit I (COI) gene, partial cds; mitochondrial

```
product length = 139
Forward primer  1    TTAATATACGGTCATTTGGTATGAGTATAGATC  33
Template        459  .................................  491

Reverse primer  1    AAGTTTCGGTCTGTTAATAATATAGTAATAGCT  33
Template        597  .................................  565
```

>HM237598.1 Charybdis japonica haplotype hap2 cytochrome oxidase subunit I (COI) gene, partial cds; mitochondrial

```
product length = 139
Forward primer  1    TTAATATACGGTCATTTGGTATGAGTATAGATC  33
Template        459  .................................  491
```

```
Reverse primer  1    AAGTTTCGGTCTGTTAATAATATAGTAATAGCT  33
Template        597  .................................  565
```

>HM237597.1 Charybdis japonica haplotype hap1 cytochrome oxidase subunit I (COI) gene, partial cds; mitochondrial

```
product length = 139
Forward primer  1    TTAATATACGGTCATTTGGTATGAGTATAGATC  33
Template        459  .................................  491

Reverse primer  1    AAGTTTCGGTCTGTTAATAATATAGTAATAGCT  33
Template        597  .................................  565
```

>EU586120.1 Charybdis japonica cytochrome oxidase subunit I (COI) gene, partial cds; mitochondrial

```
product length = 139
Forward primer  1    TTAATATACGGTCATTTGGTATGAGTATAGATC  33
Template        193  .................................  161

Reverse primer  1   AAGTTTCGGTCTGTTAATAATATAGTAATAGCT  33
Template        55  .................................  87
```

>EU284142.1 Charybdis variegata voucher LRC04 cytochrome oxidase subunit I (COI) gene, partial cds; mitochondrial

```
product length = 139
Forward primer  1    TTAATATACGGTCATTTGGTATGAGTATAGATC  33
Template        484  .................................  516

Reverse primer  1    AAGTTTCGGTCTGTTAATAATATAGTAATAGCT  33
Template        622  .................................  590
```

>FJ460517.1 Charybdis japonica mitochondrion, complete genome

```
product length = 139
Forward primer  1    TTAATATACGGTCATTTGGTATGAGTATAGATC  33
Template        500  ............................G....  532

Reverse primer  1    AAGTTTCGGTCTGTTAATAATATAGTAATAGCT  33
Template        638  ................................C  606
```

>MZ393897.1 Charybdis affinis isolate CBD15 cytochrome c oxidase subunit I (COX1) gene, partial cds; mitochondrial

```
product length = 139
Forward primer  1    TTAATATACGGTCATTTGGTATGAGTATAGATC  33
Template        459  ...................C.............  491

Reverse primer  1    AAGTTTCGGTCTGTTAATAATATAGTAATAGCT  33
Template        597  .............................G...  565
```

>MT803340.1 Charybdis japonica voucher C1910SM-11 cytochrome c oxidase subunit I (COX1) gene, partial cds; mitochondrial

```
product length = 139
Forward primer  1    TTAATATACGGTCATTTGGTATGAGTATAGATC  33
Template        363  ...................C.............  395

Reverse primer  1    AAGTTTCGGTCTGTTAATAATATAGTAATAGCT  33
Template        501  .............................G...  469
```

>MT219311.1 Charybdis japonica isolate 118371 cytochrome c oxidase subunit I (COX1) gene, partial cds; mitochondrial

```
product length = 139
Forward primer  1    TTAATATACGGTCATTTGGTATGAGTATAGATC  33
Template        399  ...................C.............  431

Reverse primer  1    AAGTTTCGGTCTGTTAATAATATAGTAATAGCT  33
Template        537  .............................G...  505
```

>MT219310.1 Charybdis japonica isolate 11837 cytochrome c oxidase subunit I (COX1) gene, partial cds; mitochondrial

```
product length = 139
Forward primer  1    TTAATATACGGTCATTTGGTATGAGTATAGATC  33
Template        427  ...................C.............  459

Reverse primer  1    AAGTTTCGGTCTGTTAATAATATAGTAATAGCT  33
Template        565  .............................G...  533
```

>OV281354.1 Marasmarcha lunaedactyla genome assembly, chromosome: 15

```
product length = 3373
Reverse primer  1         AAGTTTCGGTCTGTTAATAATATAGTAATAGCT  33
Template        20958847  ........A..A.....................  20958815

Reverse primer  1         AAGTTTCGGTCTGTTAATAATATAGTAATAGCT  33
Template        20955475  ........A..A.....................  20955507
```

>MT278110.1 Charybdis japonica voucher CC-C1901SB-07 cytochrome c oxidase subunit I (COX1) gene, partial cds; mitochondrial

```
product length = 139
Forward primer  1    TTAATATACGGTCATTTGGTATGAGTATAGATC  33
Template        392  ..........A........C.............  424

Reverse primer  1    AAGTTTCGGTCTGTTAATAATATAGTAATAGCT  33
Template        530  ...................G.........G...  498
```

>MZ393912.1 Charybdis natator isolate CBD21 cytochrome c oxidase subunit I (COX1) gene, partial cds; mitochondrial

```
product length = 139
Forward primer  1    TTAATATACGGTCATTTGGTATGAGTATAGATC  33
Template        459  ..........T..C........A........C.  491

Reverse primer  1    AAGTTTCGGTCTGTTAATAATATAGTAATAGCT  33
Template        597  ....................C............  565
```

>MT852946.1 Pseudothelphusa sulcifrons voucher CNCR 28922 cytochrome c oxidase subunit I (COX1) gene, partial cds; mitochondrial

```
product length = 139
Forward primer  1    TTAATATACGGTCATTTGGTATGAGTATAGATC  33
Template        416  ..........T........A..A.C........  448

Reverse primer  1    AAGTTTCGGTCTGTTAATAATATAGTAATAGCT  33
Template        554  ..A..............................  522
```

>MT852945.1 Pseudothelphusa belliana voucher CNCR 19228 cytochrome c oxidase subunit I (COX1) gene, partial cds; mitochondrial

```
product length = 139
Forward primer  1    TTAATATACGGTCATTTGGTATGAGTATAGATC  33
Template        416  ..........T........A..A.C........  448

Reverse primer  1    AAGTTTCGGTCTGTTAATAATATAGTAATAGCT  33
Template        554  ...................G.............  522
```

>MT852038.1 Allacanthos pittieri voucher INPA 1834 cytochrome c oxidase subunit I (COX1) gene, partial cds; mitochondrial

```
product length = 139
Forward primer  1    TTAATATACGGTCATTTGGTATGAGTATAGATC  33
Template        416  ..........T........A..A.C........  448

Reverse primer  1    AAGTTTCGGTCTGTTAATAATATAGTAATAGCT  33
Template        554  ..A..............................  522
```

>MT852036.1 Achlidon agrestis voucher UCR-MZ 3214-01 cytochrome c oxidase subunit I (COX1) gene, partial cds; mitochondrial

```
product length = 139
Forward primer  1    TTAATATACGGTCATTTGGTATGAGTATAGATC  33
Template        416  ..........T........A..A.C........  448

Reverse primer  1    AAGTTTCGGTCTGTTAATAATATAGTAATAGCT  33
Template        554  ..A..............................  522
```

>MW144286.1 Charybdis natator voucher ZSI/SRC C-168 cytochrome c oxidase subunit I (COX1) gene, partial cds; mitochondrial

```
product length = 139
Forward primer  1    TTAATATACGGTCATTTGGTATGAGTATAGATC  33
Template        437  ..........T..C........A........C.  469

Reverse primer  1    AAGTTTCGGTCTGTTAATAATATAGTAATAGCT  33
Template        575  ....................C............  543
```

>MH447071.1 Charybdis natator isolate CC cytochrome oxidase subunit 1 (COI) gene, partial cds; mitochondrial

```
product length = 139
Forward primer  1    TTAATATACGGTCATTTGGTATGAGTATAGATC  33
Template        470  ..........T..C........A........C.  502

Reverse primer  1    AAGTTTCGGTCTGTTAATAATATAGTAATAGCT  33
Template        608  ....................C............  576
```

>MH447070.1 Charybdis natator isolate CR cytochrome oxidase subunit 1 (COI) gene, partial cds; mitochondrial

```
product length = 139
Forward primer  1    TTAATATACGGTCATTTGGTATGAGTATAGATC  33
Template        440  ..........T..C........A........C.  472

Reverse primer  1    AAGTTTCGGTCTGTTAATAATATAGTAATAGCT  33
Template        578  ....................C............  546
```

>MT278106.1 Charybdis natator voucher C1903SM-04 cytochrome c oxidase subunit I (COX1) gene, partial cds; mitochondrial

```
product length = 139
Forward primer  1    TTAATATACGGTCATTTGGTATGAGTATAGATC  33
Template        392  ..........T..C........A........C.  424
```

```
Reverse primer  1    AAGTTTCGGTCTGTTAATAATATAGTAATAGCT  33
Template        530  ....................C............  498
```

>MN184693.1 Charybdis natator isolate Biosec741 cytochrome oxidase subunit I (COI) gene, partial cds; mitochondrial

```
product length = 139
Forward primer  1    TTAATATACGGTCATTTGGTATGAGTATAGATC  33
Template        459  ..........T..C........A........C.  491

Reverse primer  1    AAGTTTCGGTCTGTTAATAATATAGTAATAGCT  33
Template        597  ....................C............  565
```

>MF285241.1 Charybdis natator mitochondrion, complete genome

```
product length = 139
Forward primer  1    TTAATATACGGTCATTTGGTATGAGTATAGATC  33
Template        500  ..........T..C........A........C.  532

Reverse primer  1    AAGTTTCGGTCTGTTAATAATATAGTAATAGCT  33
Template        638  ....................C............  606
```

>KX060205.1 Charybdis natator isolate XDNA - 512 377 cytochrome oxidase subunit 1 (COI) gene, partial cds; mitochondrial

```
product length = 139
Forward primer  1    TTAATATACGGTCATTTGGTATGAGTATAGATC  33
Template        470  ..........T..C........A........C.  502

Reverse primer  1    AAGTTTCGGTCTGTTAATAATATAGTAATAGCT  33
Template        608  ....................C............  576
```

>KX060204.1 Charybdis natator isolate XDNA - 338 299 cytochrome oxidase subunit 1 (COI) gene, partial cds; mitochondrial

```
product length = 139
Forward primer  1    TTAATATACGGTCATTTGGTATGAGTATAGATC  33
Template        470  ..........T..C........A........C.  502

Reverse primer  1    AAGTTTCGGTCTGTTAATAATATAGTAATAGCT  33
Template        608  ....................C............  576
```

>MG742719.1 Charybdis granulata voucher CASMBGM-2CG cytochrome oxidase subunit 1 (COI) gene, partial cds; mitochondrial

```
product length = 139
Forward primer  1    TTAATATACGGTCATTTGGTATGAGTATAGATC  33
Template        459  ..........T..C........A........C.  491

Reverse primer  1    AAGTTTCGGTCTGTTAATAATATAGTAATAGCT  33
Template        597  ....................C............  565
```

>KT365719.1 Charybdis natator isolate FLMNH_187-E06 cytochrome oxidase subunit I (COI) gene, partial cds; mitochondrial

```
product length = 139
Forward primer  1    TTAATATACGGTCATTTGGTATGAGTATAGATC  33
Template        458  ..........T..C........A........C.  490

Reverse primer  1    AAGTTTCGGTCTGTTAATAATATAGTAATAGCT  33
Template        596  ....................C............  564
```

>KY055670.1 Allacanthos pittieri isolate R936-2 cytochrome oxidase subunit 1 (COI) gene, partial cds; mitochondrial

```
product length = 139
Forward primer  1    TTAATATACGGTCATTTGGTATGAGTATAGATC  33
Template        428  ..........T........A..A.C........  460

Reverse primer  1    AAGTTTCGGTCTGTTAATAATATAGTAATAGCT  33
Template        566  ..A..............................  534
```

>KX018513.1 Charybdis variegata strain MSCAS5 cytochrome oxidase subunit I gene, partial cds; mitochondrial

```
product length = 139
Forward primer  1    TTAATATACGGTCATTTGGTATGAGTATAGATC  33
Template        459  ..........T..C........A........C.  491

Reverse primer  1    AAGTTTCGGTCTGTTAATAATATAGTAATAGCT  33
Template        597  ....................C............  565
```

>KX381797.1 Charybdis natator voucher PCMUZ CNC 02 cytochrome oxidase subunit I (COI) gene, partial cds; mitochondrial

```
product length = 139
Forward primer  1    TTAATATACGGTCATTTGGTATGAGTATAGATC  33
Template        459  ..........T..C........A........C.  491

Reverse primer  1    AAGTTTCGGTCTGTTAATAATATAGTAATAGCT  33
Template        597  ....................C............  565
```

>KJ168053.1 Charybdis variegata voucher BTN44 cytochrome oxidase subunit 1 (COI) gene, partial cds; mitochondrial

```
product length = 139
Forward primer  1     TTAATATACGGTCATTTGGTATGAGTATAGATC  33
Template        484   ..........T..C........A........C.  516

Reverse primer  1     AAGTTTCGGTCTGTTAATAATATAGTAATAGCT  33
Template        622   ...................C............  590
```

>KF793328.1 Charybdis natator voucher Em2012-C1 cytochrome oxidase subunit I gene, partial cds; mitochondrial

```
product length = 139
Forward primer  1     TTAATATACGGTCATTTGGTATGAGTATAGATC  33
Template        270   ..........T..C........A........C.  302

Reverse primer  1     AAGTTTCGGTCTGTTAATAATATAGTAATAGCT  33
Template        408   ...................C............  376
```

>XR_007552323.1 PREDICTED: Schistocerca gregaria uncharacterized LOC126293624 (LOC126293624), transcript variant X3, misc_RNA

```
product length = 1071
Reverse primer  1      AAGTTTCGGTCTGTTAATAATATAGTAATAGCT  33
Template        1961   ..A.....A........................  1929

Reverse primer  1     AAGTTTCGGTCTGTTAATAATATAGTAATAGCT  33
Template        891   ..A.....A...C....................  923
```

>XR_007552322.1 PREDICTED: Schistocerca gregaria uncharacterized LOC126293624 (LOC126293624), transcript variant X2, misc_RNA

```
product length = 1071
Reverse primer  1      AAGTTTCGGTCTGTTAATAATATAGTAATAGCT  33
Template        1961   ..A.....A........................  1929

Reverse primer  1     AAGTTTCGGTCTGTTAATAATATAGTAATAGCT  33
Template        891   ..A.....A...C....................  923
```

>MZ393913.1 Charybdis orientalis isolate CBD16 cytochrome c oxidase subunit I (COX1) gene, partial cds; mitochondrial

```
product length = 139
Forward primer  1     TTAATATACGGTCATTTGGTATGAGTATAGATC  33
Template        459   ..........T..T........A..........  491

Reverse primer  1     AAGTTTCGGTCTGTTAATAATATAGTAATAGCT  33
Template        597   .G......A........................  565
```

>MT278109.1 Charybdis orientalis voucher C1903SM-03 cytochrome c oxidase subunit I (COX1) gene, partial cds; mitochondrial

```
product length = 139
Forward primer  1     TTAATATACGGTCATTTGGTATGAGTATAGATC  33
Template        392   ..........T..T........A..........  424

Reverse primer  1     AAGTTTCGGTCTGTTAATAATATAGTAATAGCT  33
Template        530   .G......A........................  498
```

>MW526598.1 Aedes albopictus isolate GJ_05 cytochrome c oxidase subunit I (COX1) gene, partial cds; mitochondrial

```
product length = 139
Forward primer  1     TTAATATACGGTCATTTGGTATGAGTATAGATC  33
Template        459   ..........A...GC......T.C........  491

Reverse primer  1     AAGTTTCGGTCTGTTAATAATATAGTAATAGCT  33
Template        597   ..A..............................  565
```

>MW526597.1 Aedes albopictus isolate GJ_04 cytochrome c oxidase subunit I (COX1) gene, partial cds; mitochondrial

```
product length = 139
Forward primer  1     TTAATATACGGTCATTTGGTATGAGTATAGATC  33
Template        459   ..........A...GC......T.C........  491

Reverse primer  1     AAGTTTCGGTCTGTTAATAATATAGTAATAGCT  33
Template        597   ..A..............................  565
```

>MW526596.1 Aedes albopictus isolate GJ_03 cytochrome c oxidase subunit I (COX1) gene, partial cds; mitochondrial

```
product length = 139
Forward primer  1     TTAATATACGGTCATTTGGTATGAGTATAGATC  33
Template        459   ..........A...GC......T.C........  491

Reverse primer  1     AAGTTTCGGTCTGTTAATAATATAGTAATAGCT  33
Template        597   ..A..............................  565
```

>MW526595.1 Aedes albopictus isolate GJ_02 cytochrome c oxidase subunit I (COX1) gene, partial cds; mitochondrial

```
product length = 139
Forward primer  1     TTAATATACGGTCATTTGGTATGAGTATAGATC  33
Template        459   ..........A...GC......T.C........  491
```

```
Reverse primer  1    AAGTTTCGGTCTGTTAATAATATAGTAATAGCT  33
Template        597  ..A..............................  565
```

>MW526594.1 Aedes albopictus isolate GJ_01 cytochrome c oxidase subunit I (COX1) gene, partial cds; mitochondrial

```
product length = 139
Forward primer  1    TTAATATACGGTCATTTGGTATGAGTATAGATC  33
Template        459  ..........A...GC......T.C........  491

Reverse primer  1    AAGTTTCGGTCTGTTAATAATATAGTAATAGCT  33
Template        597  ..A..............................  565
```

>OK165451.1 Pseudothelphusa doenitzi voucher CNCR 26190 cytochrome c oxidase subunit I (COX1) gene, partial cds; mitochondrial

```
product length = 139
Forward primer  1    TTAATATACGGTCATTTGGTATGAGTATAGATC  33
Template        428  ..........T........A..A.C......C.  460

Reverse primer  1    AAGTTTCGGTCTGTTAATAATATAGTAATAGCT  33
Template        566  ..................G..............  534
```

>MT852944.1 Pseudothelphusa americana voucher CNCR 25527 cytochrome c oxidase subunit I (COX1) gene, partial cds; mitochondrial

```
product length = 139
Forward primer  1    TTAATATACGGTCATTTGGTATGAGTATAGATC  33
Template        416  ..........T........A..A.C......C.  448

Reverse primer  1    AAGTTTCGGTCTGTTAATAATATAGTAATAGCT  33
Template        554  ..................G..............  522
```

>KT365743.1 Monomia petrea isolate FLMNH_143-C04 cytochrome oxidase subunit I (COI) gene, partial cds; mitochondrial

```
product length = 139
Forward primer  1    TTAATATACGGTCATTTGGTATGAGTATAGATC  33
Template        458  ....C.....C.....C.....A........C.  490

Reverse primer  1    AAGTTTCGGTCTGTTAATAATATAGTAATAGCT  33
Template        596  .....C...........................  564
```

>NC_060621.1 Charybdis hellerii mitochondrion, complete genome

```
product length = 139
Forward primer  1    TTAATATACGGTCATTTGGTATGAGTATAGATC  33
Template        500  ..........C..C........A........C.  532

Reverse primer  1    AAGTTTCGGTCTGTTAATAATATAGTAATAGCT  33
Template        638  ..A.............................G  606
```

>OM714505.1 Monomia petrea voucher MNHN-IU-2014-4071 cytochrome c oxidase subunit I (COX1) gene, partial cds; mitochondrial

```
product length = 139
Forward primer  1    TTAATATACGGTCATTTGGTATGAGTATAGATC  33
Template        416  ..........C.....C.....A........C.  448

Reverse primer  1    AAGTTTCGGTCTGTTAATAATATAGTAATAGCT  33
Template        554  .....C..........................C  522
```

>OK383006.1 Monomia petrea voucher CMLREPS11 cytochrome c oxidase subunit I (COX1) gene, partial cds; mitochondrial

```
product length = 139
Forward primer  1    TTAATATACGGTCATTTGGTATGAGTATAGATC  33
Template        314  ..........C.....C.....A........C.  346

Reverse primer  1    AAGTTTCGGTCTGTTAATAATATAGTAATAGCT  33
Template        452  .....C..........................C  420
```

>OK383005.1 Monomia petrea voucher CMLREPS7 cytochrome c oxidase subunit I (COX1) gene, partial cds; mitochondrial

```
product length = 139
Forward primer  1    TTAATATACGGTCATTTGGTATGAGTATAGATC  33
Template        314  ..........C.....C.....A........C.  346

Reverse primer  1    AAGTTTCGGTCTGTTAATAATATAGTAATAGCT  33
Template        452  .....C..........................C  420
```

>MZ393906.1 Charybdis hellerii isolate CBD19 cytochrome c oxidase subunit I (COX1) gene, partial cds; mitochondrial

```
product length = 139
Forward primer  1    TTAATATACGGTCATTTGGTATGAGTATAGATC  33
Template        459  ..........C..C........A........C.  491

Reverse primer  1    AAGTTTCGGTCTGTTAATAATATAGTAATAGCT  33
Template        597  ..A.............................A  565
```

>MW278654.1 Charybdis hellerii voucher UF:Invertebrate Zoology:45898-Arthropoda cytochrome c oxidase subunit I (COX1) gene, partial cds; mitochondrial

```
product length = 139
Forward primer  1    TTAATATACGGTCATTTGGTATGAGTATAGATC  33
Template        459  ..........C..C........A........C.  491

Reverse primer  1    AAGTTTCGGTCTGTTAATAATATAGTAATAGCT  33
Template        597  ..A.............................G  565
```

>MT852034.1 Neostrengeria macropa voucher INPA 1556 cytochrome c oxidase subunit I (COX1) gene, partial cds; mitochondrial

```
product length = 139
Forward primer  1    TTAATATACGGTCATTTGGTATGAGTATAGATC  33
Template        416  ..........T........G..A.C........  448

Reverse primer  1    AAGTTTCGGTCTGTTAATAATATAGTAATAGCT  33
Template        554  .G..............................A  522
```

>MT514358.1 Charybdis hellerii voucher DBS 008 cytochrome c oxidase subunit I (COX1) gene, partial cds; mitochondrial

```
product length = 139
Forward primer  1    TTAATATACGGTCATTTGGTATGAGTATAGATC  33
Template        405  ..........C..C........A........C.  437

Reverse primer  1    AAGTTTCGGTCTGTTAATAATATAGTAATAGCT  33
Template        543  ..A.............................G  511
```

>MK091840.1 Charybdis hellerii isolate 39A2 cytochrome c oxidase subunit I (COI) gene, partial cds; mitochondrial

```
product length = 139
Forward primer  1    TTAATATACGGTCATTTGGTATGAGTATAGATC  33
Template        469  ..........C..C........A........C.  501

Reverse primer  1    AAGTTTCGGTCTGTTAATAATATAGTAATAGCT  33
Template        607  ..A.............................G  575
```

>MK091839.1 Charybdis hellerii isolate 39A1 cytochrome c oxidase subunit I (COI) gene, partial cds; mitochondrial

```
product length = 139
Forward primer  1    TTAATATACGGTCATTTGGTATGAGTATAGATC  33
Template        469  ..........C..C........A........C.  501

Reverse primer  1    AAGTTTCGGTCTGTTAATAATATAGTAATAGCT  33
Template        607  ..A.............................G  575
```

>MK091838.1 Charybdis hellerii isolate 29A4 cytochrome c oxidase subunit I (COI) gene, partial cds; mitochondrial

```
product length = 139
Forward primer  1    TTAATATACGGTCATTTGGTATGAGTATAGATC  33
Template        469  ..........C..C........A........C.  501

Reverse primer  1    AAGTTTCGGTCTGTTAATAATATAGTAATAGCT  33
Template        607  ..A.............................G  575
```

>MK091837.1 Charybdis hellerii isolate 29A2 cytochrome c oxidase subunit I (COI) gene, partial cds; mitochondrial

```
product length = 139
Forward primer  1    TTAATATACGGTCATTTGGTATGAGTATAGATC  33
Template        469  ..........C..C........A........C.  501

Reverse primer  1    AAGTTTCGGTCTGTTAATAATATAGTAATAGCT  33
Template        607  ..A.............................G  575
```

>MK091835.1 Charybdis hellerii isolate 38A2 cytochrome c oxidase subunit I (COI) gene, partial cds; mitochondrial

```
product length = 139
Forward primer  1    TTAATATACGGTCATTTGGTATGAGTATAGATC  33
Template        469  ..........C..C........A........C.  501

Reverse primer  1    AAGTTTCGGTCTGTTAATAATATAGTAATAGCT  33
Template        607  ..A.............................G  575
```

>MN184692.1 Charybdis hellerii isolate Biosec894 cytochrome oxidase subunit I (COI) gene, partial cds; mitochondrial

```
product length = 139
Forward primer  1    TTAATATACGGTCATTTGGTATGAGTATAGATC  33
Template        459  ..........C..C........A........C.  491

Reverse primer  1    AAGTTTCGGTCTGTTAATAATATAGTAATAGCT  33
Template        597  ..A.............................G  565
```

>MN811221.1 Charybdis hellerii voucher USNM1408295 cytochrome c oxidase subunit I (COX1) gene, partial cds; mitochondrial

```
product length = 139
```

```
Forward primer  1    TTAATATACGGTCATTTGGTATGAGTATAGATC  33
Template        459  ..........C..C........A........C.  491

Reverse primer  1    AAGTTTCGGTCTGTTAATAATATAGTAATAGCT  33
Template        597  ..A.............................G  565
```

>MG729773.1 Potamon transcaspicum voucher SMF:24233 cytochrome oxidase subunit I (COI) gene, partial cds; mitochondrial

```
product length = 139
Forward primer  1    TTAATATACGGTCATTTGGTATGAGTATAGATC  33
Template        440  ..........T..T........A.C........  472

Reverse primer  1    AAGTTTCGGTCTGTTAATAATATAGTAATAGCT  33
Template        578  ........A.......................C  546
```

>MF693858.1 Charybdis hellerii isolate NESEP09F02 cytochrome oxidase subunit I (COI) gene, partial cds; mitochondrial

```
product length = 139
Forward primer  1    TTAATATACGGTCATTTGGTATGAGTATAGATC  33
Template        459  ..........C..C........A........C.  491

Reverse primer  1    AAGTTTCGGTCTGTTAATAATATAGTAATAGCT  33
Template        597  ..A.............................G  565
```

>KT365715.1 Charybdis hellerii isolate E070 cytochrome oxidase subunit I (COI) gene, partial cds; mitochondrial

```
product length = 139
Forward primer  1    TTAATATACGGTCATTTGGTATGAGTATAGATC  33
Template        458  ..........C..C........A........C.  490

Reverse primer  1    AAGTTTCGGTCTGTTAATAATATAGTAATAGCT  33
Template        596  ..A.............................G  564
```

>KX060356.1 Charybdis hellerii isolate XDNA - 638 485 cytochrome oxidase subunit 1 (COI) gene, partial cds; mitochondrial

```
product length = 139
Forward primer  1    TTAATATACGGTCATTTGGTATGAGTATAGATC  33
Template        380  ..........C..C........A........C.  412

Reverse primer  1    AAGTTTCGGTCTGTTAATAATATAGTAATAGCT  33
Template        518  ..A.............................G  486
```

>KX060355.1 Charybdis hellerii isolate XDNA - 638 484 cytochrome oxidase subunit 1 (COI) gene, partial cds; mitochondrial

```
product length = 139
Forward primer  1    TTAATATACGGTCATTTGGTATGAGTATAGATC  33
Template        473  ..........C..C........A........C.  505

Reverse primer  1    AAGTTTCGGTCTGTTAATAATATAGTAATAGCT  33
Template        611  ..A.............................G  579
```

>KX060354.1 Charybdis hellerii isolate XDNA - 638 483 cytochrome oxidase subunit 1 (COI) gene, partial cds; mitochondrial

```
product length = 139
Forward primer  1    TTAATATACGGTCATTTGGTATGAGTATAGATC  33
Template        446  ..........C..C........A........C.  478

Reverse primer  1    AAGTTTCGGTCTGTTAATAATATAGTAATAGCT  33
Template        584  ..A.............................G  552
```

>KX060352.1 Charybdis hellerii isolate XDNA - 638 463 cytochrome oxidase subunit 1 (COI) gene, partial cds; mitochondrial

```
product length = 139
Forward primer  1    TTAATATACGGTCATTTGGTATGAGTATAGATC  33
Template        446  ..........C..C........A........C.  478

Reverse primer  1    AAGTTTCGGTCTGTTAATAATATAGTAATAGCT  33
Template        584  ..A.............................G  552
```

>KX060350.1 Charybdis hellerii isolate XDNA - 638 461 cytochrome oxidase subunit 1 (COI) gene, partial cds; mitochondrial

```
product length = 139
Forward primer  1    TTAATATACGGTCATTTGGTATGAGTATAGATC  33
Template        443  ..........C..C........A........C.  475

Reverse primer  1    AAGTTTCGGTCTGTTAATAATATAGTAATAGCT  33
Template        581  ..A.............................G  549
```

>KX060339.1 Charybdis hellerii isolate XDNA - 1039 434 cytochrome oxidase subunit 1 (COI) gene, partial cds; mitochondrial

```
product length = 139
Forward primer  1    TTAATATACGGTCATTTGGTATGAGTATAGATC  33
Template        473  ..........C..C........A........C.  505

Reverse primer  1    AAGTTTCGGTCTGTTAATAATATAGTAATAGCT  33
```

```
Template        611  ..A.............................G  579
```

>KX060337.1 Charybdis hellerii isolate XDNA - 1039 426 cytochrome oxidase subunit 1 (COI) gene, partial cds; mitochondrial

```
product length = 139
Forward primer  1    TTAATATACGGTCATTTGGTATGAGTATAGATC  33
Template        443  ..........C..C........A........C.  475

Reverse primer  1    AAGTTTCGGTCTGTTAATAATATAGTAATAGCT  33
Template        581  ..A.............................G  549
```

>KX060331.1 Charybdis hellerii isolate XDNA - 1039 419 cytochrome oxidase subunit 1 (COI) gene, partial cds; mitochondrial

```
product length = 139
Forward primer  1    TTAATATACGGTCATTTGGTATGAGTATAGATC  33
Template        473  ..........C..C........A........C.  505

Reverse primer  1    AAGTTTCGGTCTGTTAATAATATAGTAATAGCT  33
Template        611  ..A.............................G  579
```

>KX060328.1 Charybdis hellerii isolate XDNA - 1039 406 cytochrome oxidase subunit 1 (COI) gene, partial cds; mitochondrial

```
product length = 139
Forward primer  1    TTAATATACGGTCATTTGGTATGAGTATAGATC  33
Template        386  ..........C..C........A........C.  418

Reverse primer  1    AAGTTTCGGTCTGTTAATAATATAGTAATAGCT  33
Template        524  ..A.............................G  492
```

>KX060325.1 Charybdis hellerii isolate XDNA - 506 366 cytochrome oxidase subunit 1 (COI) gene, partial cds; mitochondrial

```
product length = 139
Forward primer  1    TTAATATACGGTCATTTGGTATGAGTATAGATC  33
Template        473  ..........C..C........A........C.  505

Reverse primer  1    AAGTTTCGGTCTGTTAATAATATAGTAATAGCT  33
Template        611  ..A.............................G  579
```

>KX060322.1 Charybdis hellerii isolate XDNA - 1039 363 cytochrome oxidase subunit 1 (COI) gene, partial cds; mitochondrial

```
product length = 139
Forward primer  1    TTAATATACGGTCATTTGGTATGAGTATAGATC  33
Template        473  ..........C..C........A........C.  505

Reverse primer  1    AAGTTTCGGTCTGTTAATAATATAGTAATAGCT  33
Template        611  ..A.............................G  579
```

>KX060321.1 Charybdis hellerii isolate XDNA - 1039 362 cytochrome oxidase subunit 1 (COI) gene, partial cds; mitochondrial

```
product length = 139
Forward primer  1    TTAATATACGGTCATTTGGTATGAGTATAGATC  33
Template        473  ..........C..C........A........C.  505

Reverse primer  1    AAGTTTCGGTCTGTTAATAATATAGTAATAGCT  33
Template        611  ..A.............................G  579
```

>KX060319.1 Charybdis hellerii isolate XDNA - 1039 360 cytochrome oxidase subunit 1 (COI) gene, partial cds; mitochondrial

```
product length = 139
Forward primer  1    TTAATATACGGTCATTTGGTATGAGTATAGATC  33
Template        473  ..........C..C........A........C.  505

Reverse primer  1    AAGTTTCGGTCTGTTAATAATATAGTAATAGCT  33
Template        611  ..A.............................G  579
```

>KX060318.1 Charybdis hellerii isolate XDNA - 506 345 cytochrome oxidase subunit 1 (COI) gene, partial cds; mitochondrial

```
product length = 139
Forward primer  1    TTAATATACGGTCATTTGGTATGAGTATAGATC  33
Template        473  ..........C..C........A........C.  505

Reverse primer  1    AAGTTTCGGTCTGTTAATAATATAGTAATAGCT  33
Template        611  ..A.............................G  579
```

>KX060317.1 Charybdis hellerii isolate XDNA - 1039 344 cytochrome oxidase subunit 1 (COI) gene, partial cds; mitochondrial

```
product length = 139
Forward primer  1    TTAATATACGGTCATTTGGTATGAGTATAGATC  33
Template        473  ..........C..C........A........C.  505

Reverse primer  1    AAGTTTCGGTCTGTTAATAATATAGTAATAGCT  33
Template        611  ..A.............................G  579
```

>KX060316.1 Charybdis hellerii isolate XDNA - 1039 343 cytochrome oxidase subunit 1 (COI) gene, partial cds; mitochondrial

```
product length = 139
Forward primer  1    TTAATATACGGTCATTTGGTATGAGTATAGATC  33
Template        446  .........C..C........A........C.  478

Reverse primer  1    AAGTTTCGGTCTGTTAATAATATAGTAATAGCT  33
Template        584  ..A.............................G  552
```

>KX060315.1 Charybdis hellerii isolate XDNA - 1039 342 cytochrome oxidase subunit 1 (COI) gene, partial cds; mitochondrial

```
product length = 139
Forward primer  1    TTAATATACGGTCATTTGGTATGAGTATAGATC  33
Template        473  .........C..C........A........C.  505

Reverse primer  1    AAGTTTCGGTCTGTTAATAATATAGTAATAGCT  33
Template        611  ..A.............................A  579
```

>KX060314.1 Charybdis hellerii isolate XDNA - 1039 341 cytochrome oxidase subunit 1 (COI) gene, partial cds; mitochondrial

```
product length = 139
Forward primer  1    TTAATATACGGTCATTTGGTATGAGTATAGATC  33
Template        473  .........C..C........A........C.  505

Reverse primer  1    AAGTTTCGGTCTGTTAATAATATAGTAATAGCT  33
Template        611  ..A.............................G  579
```

>KX060313.1 Charybdis hellerii isolate XDNA - 1039 340 cytochrome oxidase subunit 1 (COI) gene, partial cds; mitochondrial

```
product length = 139
Forward primer  1    TTAATATACGGTCATTTGGTATGAGTATAGATC  33
Template        473  .........C..C........A........C.  505

Reverse primer  1    AAGTTTCGGTCTGTTAATAATATAGTAATAGCT  33
Template        611  ..A.............................G  579
```

>KX060312.1 Charybdis hellerii isolate XDNA - 377 322 cytochrome oxidase subunit 1 (COI) gene, partial cds; mitochondrial

```
product length = 139
Forward primer  1    TTAATATACGGTCATTTGGTATGAGTATAGATC  33
Template        446  .........C..C........A........C.  478

Reverse primer  1    AAGTTTCGGTCTGTTAATAATATAGTAATAGCT  33
Template        584  ..A.............................G  552
```

>KX060311.1 Charybdis hellerii isolate XDNA - 377 321 cytochrome oxidase subunit 1 (COI) gene, partial cds; mitochondrial

```
product length = 139
Forward primer  1    TTAATATACGGTCATTTGGTATGAGTATAGATC  33
Template        470  .........C..C........A........C.  502

Reverse primer  1    AAGTTTCGGTCTGTTAATAATATAGTAATAGCT  33
Template        608  ..A.............................G  576
```

>KX060309.1 Charybdis hellerii isolate XDNA - 367 316 cytochrome oxidase subunit 1 (COI) gene, partial cds; mitochondrial

```
product length = 139
Forward primer  1    TTAATATACGGTCATTTGGTATGAGTATAGATC  33
Template        452  .........C..C........A........C.  484

Reverse primer  1    AAGTTTCGGTCTGTTAATAATATAGTAATAGCT  33
Template        590  ..A.............................G  558
```

>KX060308.1 Charybdis hellerii isolate XDNA - 367 315 cytochrome oxidase subunit 1 (COI) gene, partial cds; mitochondrial

```
product length = 139
Forward primer  1    TTAATATACGGTCATTTGGTATGAGTATAGATC  33
Template        473  .........C..C........A........C.  505

Reverse primer  1    AAGTTTCGGTCTGTTAATAATATAGTAATAGCT  33
Template        611  ..A.............................G  579
```

>KX060307.1 Charybdis hellerii isolate XDNA - 367 314 cytochrome oxidase subunit 1 (COI) gene, partial cds; mitochondrial

```
product length = 139
Forward primer  1    TTAATATACGGTCATTTGGTATGAGTATAGATC  33
Template        473  .........C..C........A........C.  505

Reverse primer  1    AAGTTTCGGTCTGTTAATAATATAGTAATAGCT  33
Template        611  ..A.............................G  579
```

>KX060306.1 Charybdis hellerii isolate XDNA - 367 313 cytochrome oxidase subunit 1 (COI) gene, partial cds; mitochondrial

```
product length = 139
Forward primer  1    TTAATATACGGTCATTTGGTATGAGTATAGATC  33
Template        443  .........C..C........A........C.  475

Reverse primer  1    AAGTTTCGGTCTGTTAATAATATAGTAATAGCT  33
```

```
Template        581  ..A............................G  549
```

>KX060305.1 Charybdis hellerii isolate XDNA - 367 312 cytochrome oxidase subunit 1 (COI) gene, partial cds; mitochondrial

```
product length = 139
Forward primer  1    TTAATATACGGTCATTTGGTATGAGTATAGATC  33
Template        449  ..........C..C........A........C.  481

Reverse primer  1    AAGTTTCGGTCTGTTAATAATATAGTAATAGCT  33
Template        587  ..A............................G  555
```

>KX060304.1 Charybdis hellerii isolate XDNA - 367 311 cytochrome oxidase subunit 1 (COI) gene, partial cds; mitochondrial

```
product length = 139
Forward primer  1    TTAATATACGGTCATTTGGTATGAGTATAGATC  33
Template        446  ..........C..C........A........C.  478

Reverse primer  1    AAGTTTCGGTCTGTTAATAATATAGTAATAGCT  33
Template        584  ..A............................G  552
```

>KX060301.1 Charybdis hellerii isolate XDNA - 338 280 cytochrome oxidase subunit 1 (COI) gene, partial cds; mitochondrial

```
product length = 139
Forward primer  1    TTAATATACGGTCATTTGGTATGAGTATAGATC  33
Template        443  ..........C..C........A........C.  475

Reverse primer  1    AAGTTTCGGTCTGTTAATAATATAGTAATAGCT  33
Template        581  ..A............................G  549
```

>KX060299.1 Charybdis hellerii isolate XDNA - 338 278 cytochrome oxidase subunit 1 (COI) gene, partial cds; mitochondrial

```
product length = 139
Forward primer  1    TTAATATACGGTCATTTGGTATGAGTATAGATC  33
Template        473  ..........C..C........A........C.  505

Reverse primer  1    AAGTTTCGGTCTGTTAATAATATAGTAATAGCT  33
Template        611  ..A............................G  579
```

>KX060296.1 Charybdis hellerii isolate XDNA - 338 275 cytochrome oxidase subunit 1 (COI) gene, partial cds; mitochondrial

```
product length = 139
Forward primer  1    TTAATATACGGTCATTTGGTATGAGTATAGATC  33
Template        383  ..........C..C........A........C.  415

Reverse primer  1    AAGTTTCGGTCTGTTAATAATATAGTAATAGCT  33
Template        521  ..A............................G  489
```

>KX060295.1 Charybdis hellerii isolate XDNA - 338 274 cytochrome oxidase subunit 1 (COI) gene, partial cds; mitochondrial

```
product length = 139
Forward primer  1    TTAATATACGGTCATTTGGTATGAGTATAGATC  33
Template        473  ..........C..C........A........C.  505

Reverse primer  1    AAGTTTCGGTCTGTTAATAATATAGTAATAGCT  33
Template        611  ..A............................G  579
```

>KX060292.1 Charybdis hellerii isolate XDNA - 366 246 cytochrome oxidase subunit 1 (COI) gene, partial cds; mitochondrial

```
product length = 139
Forward primer  1    TTAATATACGGTCATTTGGTATGAGTATAGATC  33
Template        383  ..........C..C........A........C.  415

Reverse primer  1    AAGTTTCGGTCTGTTAATAATATAGTAATAGCT  33
Template        521  ..A............................G  489
```

>KX060289.1 Charybdis hellerii isolate XDNA - 316 223 cytochrome oxidase subunit 1 (COI) gene, partial cds; mitochondrial

```
product length = 139
Forward primer  1    TTAATATACGGTCATTTGGTATGAGTATAGATC  33
Template        446  ..........C..C........A........C.  478

Reverse primer  1    AAGTTTCGGTCTGTTAATAATATAGTAATAGCT  33
Template        584  ..A............................G  552
```

>KX060285.1 Charybdis hellerii isolate XDNA - 275 218 cytochrome oxidase subunit 1 (COI) gene, partial cds; mitochondrial

```
product length = 139
Forward primer  1    TTAATATACGGTCATTTGGTATGAGTATAGATC  33
Template        470  ..........C..C........A........C.  502

Reverse primer  1    AAGTTTCGGTCTGTTAATAATATAGTAATAGCT  33
Template        608  ..A............................G  576
```

>KX060284.1 Charybdis hellerii isolate XDNA - 275 217 cytochrome oxidase subunit 1 (COI) gene, partial cds; mitochondrial

```
product length = 139
Forward primer  1    TTAATATACGGTCATTTGGTATGAGTATAGATC  33
Template        449  .........C..C........A........C.  481

Reverse primer  1    AAGTTTCGGTCTGTTAATAATATAGTAATAGCT  33
Template        587  ..A............................G  555
```

>KX060283.1 Charybdis hellerii isolate XDNA - 275 216 cytochrome oxidase subunit 1 (COI) gene, partial cds; mitochondrial

```
product length = 139
Forward primer  1    TTAATATACGGTCATTTGGTATGAGTATAGATC  33
Template        470  .........C..C........A........C.  502

Reverse primer  1    AAGTTTCGGTCTGTTAATAATATAGTAATAGCT  33
Template        608  ..A............................G  576
```

>KX060282.1 Charybdis hellerii isolate XDNA - 275 215 cytochrome oxidase subunit 1 (COI) gene, partial cds; mitochondrial

```
product length = 139
Forward primer  1    TTAATATACGGTCATTTGGTATGAGTATAGATC  33
Template        470  .........C..C........A........C.  502

Reverse primer  1    AAGTTTCGGTCTGTTAATAATATAGTAATAGCT  33
Template        608  ..A............................G  576
```

>KX060281.1 Charybdis hellerii isolate XDNA - 275 214 cytochrome oxidase subunit 1 (COI) gene, partial cds; mitochondrial

```
product length = 139
Forward primer  1    TTAATATACGGTCATTTGGTATGAGTATAGATC  33
Template        467  .........C..C........A........C.  499

Reverse primer  1    AAGTTTCGGTCTGTTAATAATATAGTAATAGCT  33
Template        605  ..A............................G  573
```

>KX060280.1 Charybdis hellerii isolate XDNA - 275 212 cytochrome oxidase subunit 1 (COI) gene, partial cds; mitochondrial

```
product length = 139
Forward primer  1    TTAATATACGGTCATTTGGTATGAGTATAGATC  33
Template        473  .........C..C........A........C.  505

Reverse primer  1    AAGTTTCGGTCTGTTAATAATATAGTAATAGCT  33
Template        611  ..A............................G  579
```

>KX060279.1 Charybdis hellerii isolate XDNA - 275 211 cytochrome oxidase subunit 1 (COI) gene, partial cds; mitochondrial

```
product length = 139
Forward primer  1    TTAATATACGGTCATTTGGTATGAGTATAGATC  33
Template        449  .........C..C........A........C.  481

Reverse primer  1    AAGTTTCGGTCTGTTAATAATATAGTAATAGCT  33
Template        587  ..A............................G  555
```

>KX060277.1 Charybdis hellerii isolate XDNA - 230 189 cytochrome oxidase subunit 1 (COI) gene, partial cds; mitochondrial

```
product length = 139
Forward primer  1    TTAATATACGGTCATTTGGTATGAGTATAGATC  33
Template        449  .........C..C........A........C.  481

Reverse primer  1    AAGTTTCGGTCTGTTAATAATATAGTAATAGCT  33
Template        587  ..A............................G  555
```

>KX060276.1 Charybdis hellerii isolate XDNA - 230 188 cytochrome oxidase subunit 1 (COI) gene, partial cds; mitochondrial

```
product length = 139
Forward primer  1    TTAATATACGGTCATTTGGTATGAGTATAGATC  33
Template        467  .........C..C........A........C.  499

Reverse primer  1    AAGTTTCGGTCTGTTAATAATATAGTAATAGCT  33
Template        605  ..A............................G  573
```

>KX060275.1 Charybdis hellerii isolate XDNA - 230 187 cytochrome oxidase subunit 1 (COI) gene, partial cds; mitochondrial

```
product length = 139
Forward primer  1    TTAATATACGGTCATTTGGTATGAGTATAGATC  33
Template        467  .........C..C........A........C.  499

Reverse primer  1    AAGTTTCGGTCTGTTAATAATATAGTAATAGCT  33
Template        605  ..A............................G  573
```

>KX060271.1 Charybdis hellerii isolate XDNA - 230 180 cytochrome oxidase subunit 1 (COI) gene, partial cds; mitochondrial

```
product length = 139
Forward primer  1    TTAATATACGGTCATTTGGTATGAGTATAGATC  33
Template        473  .........C..C........A........C.  505

Reverse primer  1    AAGTTTCGGTCTGTTAATAATATAGTAATAGCT  33
```

```
Template        611  ..A............................G  579
```

>KX060270.1 Charybdis hellerii isolate XDNA - 230 179 cytochrome oxidase subunit 1 (COI) gene, partial cds; mitochondrial

```
product length = 139
Forward primer  1    TTAATATACGGTCATTTGGTATGAGTATAGATC  33
Template        449  ..........C..C........A........C.  481

Reverse primer  1    AAGTTTCGGTCTGTTAATAATATAGTAATAGCT  33
Template        587  ..A............................G  555
```

>KX060268.1 Charybdis hellerii isolate XDNA - 230 176 cytochrome oxidase subunit 1 (COI) gene, partial cds; mitochondrial

```
product length = 139
Forward primer  1    TTAATATACGGTCATTTGGTATGAGTATAGATC  33
Template        464  ..........C..C........A........C.  496

Reverse primer  1    AAGTTTCGGTCTGTTAATAATATAGTAATAGCT  33
Template        602  ..A............................G  570
```

>KX060266.1 Charybdis hellerii isolate XDNA - 229 172 cytochrome oxidase subunit 1 (COI) gene, partial cds; mitochondrial

```
product length = 139
Forward primer  1    TTAATATACGGTCATTTGGTATGAGTATAGATC  33
Template        449  ..........C..C........A........C.  481

Reverse primer  1    AAGTTTCGGTCTGTTAATAATATAGTAATAGCT  33
Template        587  ..A............................G  555
```

>KX060265.1 Charybdis hellerii isolate XDNA - 229 167 cytochrome oxidase subunit 1 (COI) gene, partial cds; mitochondrial

```
product length = 139
Forward primer  1    TTAATATACGGTCATTTGGTATGAGTATAGATC  33
Template        446  ..........C..C........A........C.  478

Reverse primer  1    AAGTTTCGGTCTGTTAATAATATAGTAATAGCT  33
Template        584  ..A............................G  552
```

>KX060263.1 Charybdis hellerii isolate XDNA - 197 143 cytochrome oxidase subunit 1 (COI) gene, partial cds; mitochondrial

```
product length = 139
Forward primer  1    TTAATATACGGTCATTTGGTATGAGTATAGATC  33
Template        473  ..........C..C........A........C.  505

Reverse primer  1    AAGTTTCGGTCTGTTAATAATATAGTAATAGCT  33
Template        611  ..A............................G  579
```

>KX060260.1 Charybdis hellerii isolate XDNA - 229 166 cytochrome oxidase subunit 1 (COI) gene, partial cds; mitochondrial

```
product length = 139
Forward primer  1    TTAATATACGGTCATTTGGTATGAGTATAGATC  33
Template        473  ..........C..C........A........C.  505

Reverse primer  1    AAGTTTCGGTCTGTTAATAATATAGTAATAGCT  33
Template        611  ..A............................G  579
```

>KX060259.1 Charybdis hellerii isolate XDNA - 229 164 cytochrome oxidase subunit 1 (COI) gene, partial cds; mitochondrial

```
product length = 139
Forward primer  1    TTAATATACGGTCATTTGGTATGAGTATAGATC  33
Template        470  ..........C..C........A........C.  502

Reverse primer  1    AAGTTTCGGTCTGTTAATAATATAGTAATAGCT  33
Template        608  ..A............................G  576
```

>KX060257.1 Charybdis hellerii isolate XDNA - 233 196 cytochrome oxidase subunit 1 (COI) gene, partial cds; mitochondrial

```
product length = 139
Forward primer  1    TTAATATACGGTCATTTGGTATGAGTATAGATC  33
Template        464  ..........C..C........A........C.  496

Reverse primer  1    AAGTTTCGGTCTGTTAATAATATAGTAATAGCT  33
Template        602  ..A............................G  570
```

>KX060255.1 Charybdis hellerii isolate XDNA - 208 156 cytochrome oxidase subunit 1 (COI) gene, partial cds; mitochondrial

```
product length = 139
Forward primer  1    TTAATATACGGTCATTTGGTATGAGTATAGATC  33
Template        473  ..........C..C........A........C.  505

Reverse primer  1    AAGTTTCGGTCTGTTAATAATATAGTAATAGCT  33
Template        611  ..A............................G  579
```

>KX060252.1 Charybdis hellerii isolate XDNA - 208 152 cytochrome oxidase subunit 1 (COI) gene, partial cds; mitochondrial

```
product length = 139
Forward primer  1    TTAATATACGGTCATTTGGTATGAGTATAGATC  33
Template        464  .........C..C........A........C.  496

Reverse primer  1    AAGTTTCGGTCTGTTAATAATATAGTAATAGCT  33
Template        602  ..A............................G  570
```

>KX060248.1 Charybdis hellerii isolate XDNA - 197 147 cytochrome oxidase subunit 1 (COI) gene, partial cds; mitochondrial

```
product length = 139
Forward primer  1    TTAATATACGGTCATTTGGTATGAGTATAGATC  33
Template        470  .........C..C........A........C.  502

Reverse primer  1    AAGTTTCGGTCTGTTAATAATATAGTAATAGCT  33
Template        608  ..A............................G  576
```

>KX060247.1 Charybdis hellerii isolate XDNA - 197 146 cytochrome oxidase subunit 1 (COI) gene, partial cds; mitochondrial

```
product length = 139
Forward primer  1    TTAATATACGGTCATTTGGTATGAGTATAGATC  33
Template        449  .........C..C........A........C.  481

Reverse primer  1    AAGTTTCGGTCTGTTAATAATATAGTAATAGCT  33
Template        587  ..A............................G  555
```

>KX060246.1 Charybdis hellerii isolate XDNA - 187 135 cytochrome oxidase subunit 1 (COI) gene, partial cds; mitochondrial

```
product length = 139
Forward primer  1    TTAATATACGGTCATTTGGTATGAGTATAGATC  33
Template        467  .........C..C........A........C.  499

Reverse primer  1    AAGTTTCGGTCTGTTAATAATATAGTAATAGCT  33
Template        605  ..A............................G  573
```

>KX060245.1 Charybdis hellerii isolate XDNA - 187 134 cytochrome oxidase subunit 1 (COI) gene, partial cds; mitochondrial

```
product length = 139
Forward primer  1    TTAATATACGGTCATTTGGTATGAGTATAGATC  33
Template        464  .........C..C........A........C.  496

Reverse primer  1    AAGTTTCGGTCTGTTAATAATATAGTAATAGCT  33
Template        602  ..A............................G  570
```

>KX060238.1 Charybdis hellerii isolate XDNA - 187 123 cytochrome oxidase subunit 1 (COI) gene, partial cds; mitochondrial

```
product length = 139
Forward primer  1    TTAATATACGGTCATTTGGTATGAGTATAGATC  33
Template        473  .........C..C........A........C.  505

Reverse primer  1    AAGTTTCGGTCTGTTAATAATATAGTAATAGCT  33
Template        611  ..A............................G  579
```

>KX060234.1 Charybdis hellerii isolate XDNA - 187 118 cytochrome oxidase subunit 1 (COI) gene, partial cds; mitochondrial

```
product length = 139
Forward primer  1    TTAATATACGGTCATTTGGTATGAGTATAGATC  33
Template        473  .........C..C........A........C.  505

Reverse primer  1    AAGTTTCGGTCTGTTAATAATATAGTAATAGCT  33
Template        611  ..A............................G  579
```

>KX060233.1 Charybdis hellerii isolate XDNA - 187 117 cytochrome oxidase subunit 1 (COI) gene, partial cds; mitochondrial

```
product length = 139
Forward primer  1    TTAATATACGGTCATTTGGTATGAGTATAGATC  33
Template        473  .........C..C........A........C.  505

Reverse primer  1    AAGTTTCGGTCTGTTAATAATATAGTAATAGCT  33
Template        611  ..A............................G  579
```

>KX060230.1 Charybdis hellerii isolate XDNA - 172 112 cytochrome oxidase subunit 1 (COI) gene, partial cds; mitochondrial

```
product length = 139
Forward primer  1    TTAATATACGGTCATTTGGTATGAGTATAGATC  33
Template        473  .........C..C........A........C.  505

Reverse primer  1    AAGTTTCGGTCTGTTAATAATATAGTAATAGCT  33
Template        611  ..A............................G  579
```

>KX060229.1 Charybdis hellerii isolate XDNA - 172 110 cytochrome oxidase subunit 1 (COI) gene, partial cds; mitochondrial

```
product length = 139
Forward primer  1    TTAATATACGGTCATTTGGTATGAGTATAGATC  33
Template        470  .........C..C........A........C.  502

Reverse primer  1    AAGTTTCGGTCTGTTAATAATATAGTAATAGCT  33
```

```
Template        608   ..A............................G  576
```

>KX060226.1 Charybdis hellerii isolate XDNA - 172 107 cytochrome oxidase subunit 1 (COI) gene, partial cds; mitochondrial

```
product length = 139
Forward primer  1     TTAATATACGGTCATTTGGTATGAGTATAGATC  33
Template        473   .........C..C........A........C.  505

Reverse primer  1     AAGTTTCGGTCTGTTAATAATATAGTAATAGCT  33
Template        611   ..A............................G  579
```

>KX060225.1 Charybdis hellerii isolate XDNA - 172 106 cytochrome oxidase subunit 1 (COI) gene, partial cds; mitochondrial

```
product length = 139
Forward primer  1     TTAATATACGGTCATTTGGTATGAGTATAGATC  33
Template        470   .........C..C........A........C.  502

Reverse primer  1     AAGTTTCGGTCTGTTAATAATATAGTAATAGCT  33
Template        608   ..A............................G  576
```

>KX060224.1 Charybdis hellerii isolate XDNA - 172 105 cytochrome oxidase subunit 1 (COI) gene, partial cds; mitochondrial

```
product length = 139
Forward primer  1     TTAATATACGGTCATTTGGTATGAGTATAGATC  33
Template        473   .........C..C........A........C.  505

Reverse primer  1     AAGTTTCGGTCTGTTAATAATATAGTAATAGCT  33
Template        611   ..A............................G  579
```

>KX060223.1 Charybdis hellerii isolate XDNA - 172 104 cytochrome oxidase subunit 1 (COI) gene, partial cds; mitochondrial

```
product length = 139
Forward primer  1     TTAATATACGGTCATTTGGTATGAGTATAGATC  33
Template        464   .........C..C........A........C.  496

Reverse primer  1     AAGTTTCGGTCTGTTAATAATATAGTAATAGCT  33
Template        602   ..A............................G  570
```

>KU578956.1 Neostrengeria macropa isolate SMF20034 cytochrome oxidase subunit 1 (COI) gene, partial cds; mitochondrial

```
product length = 139
Forward primer  1     TTAATATACGGTCATTTGGTATGAGTATAGATC  33
Template        458   .........T........G..A.C........  490

Reverse primer  1     AAGTTTCGGTCTGTTAATAATATAGTAATAGCT  33
Template        596   .G.............................A  564
```

>KX018512.1 Charybdis hellerii strain MSCAS4 cytochrome oxidase subunit I gene, partial cds; mitochondrial

```
product length = 139
Forward primer  1     TTAATATACGGTCATTTGGTATGAGTATAGATC  33
Template        459   .........C..C........A........C.  491

Reverse primer  1     AAGTTTCGGTCTGTTAATAATATAGTAATAGCT  33
Template        597   ..A............................G  565
```

>KP254353.1 Charybdis hellerii voucher FTP_0071 cytochrome oxidase subunit 1 (COI) gene, partial cds; mitochondrial

```
product length = 139
Forward primer  1     TTAATATACGGTCATTTGGTATGAGTATAGATC  33
Template        459   .........C..C........A........C.  491

Reverse primer  1     AAGTTTCGGTCTGTTAATAATATAGTAATAGCT  33
Template        597   ..A............................G  565
```

>MN184097.1 Charybdis hellerii voucher ULLZ13465 cytochrome oxidase subunit 1 (COI) gene, partial cds; mitochondrial

```
product length = 139
Forward primer  1     TTAATATACGGTCATTTGGTATGAGTATAGATC  33
Template        459   .........C..C........A........C.  491

Reverse primer  1     AAGTTTCGGTCTGTTAATAATATAGTAATAGCT  33
Template        597   ..A............................G  565
```

>KF714930.1 Decapoda sp. BOLD:AAO9264 voucher CrP52 cytochrome oxidase subunit 1 (COI) gene, partial cds; mitochondrial

```
product length = 139
Forward primer  1     TTAATATACGGTCATTTGGTATGAGTATAGATC  33
Template        467   .........C..C........A........C.  499

Reverse primer  1     AAGTTTCGGTCTGTTAATAATATAGTAATAGCT  33
Template        605   ..A............................G  573
```

>KF870477.1 Charybdis hellerii voucher CHIpC1 cytochrome oxidase subunit I gene, partial cds; mitochondrial

```
product length = 139
Forward primer  1     TTAATATACGGTCATTTGGTATGAGTATAGATC  33
Template        421   .........C..C........A........C.  453

Reverse primer  1     AAGTTTCGGTCTGTTAATAATATAGTAATAGCT  33
Template        559   ..A.............................G  527
```

>KF793329.1 Charybdis hellerii voucher Em2012-C2 cytochrome oxidase subunit I gene, partial cds; mitochondrial

```
product length = 139
Forward primer  1     TTAATATACGGTCATTTGGTATGAGTATAGATC  33
Template        459   .........C..C........A........C.  491

Reverse primer  1     AAGTTTCGGTCTGTTAATAATATAGTAATAGCT  33
Template        597   ..A.............................G  565
```

>MW264448.1 Charybdis hellerii voucher MEGML10 cytochrome c oxidase subunit I (COX1) gene, partial cds; mitochondrial

```
product length = 139
Forward primer  1     TTAATATACGGTCATTTGGTATGAGTATAGATC  33
Template        463   .........C..C........A........C.  495

Reverse primer  1     AAGTTTCGGTCTGTTAATAATATAGTAATAGCT  33
Template        601   ..A.............................G  569
```

>MZ560548.1 Chlorodiella corallicola voucher USNM:1466898 cytochrome c oxidase subunit I (COX1) gene, partial cds; mitochondrial

```
product length = 139
Forward primer  1     TTAATATACGGTCATTTGGTATGAGTATAGATC  33
Template        459   .........C........A....C...G....  491

Reverse primer  1     AAGTTTCGGTCTGTTAATAATATAGTAATAGCT  33
Template        597   .GA.............................  565
```

>MZ560506.1 Chlorodiella corallicola voucher USNM:1466900 cytochrome c oxidase subunit I (COX1) gene, partial cds; mitochondrial

```
product length = 139
Forward primer  1     TTAATATACGGTCATTTGGTATGAGTATAGATC  33
Template        459   .........C........A....C...G....  491

Reverse primer  1     AAGTTTCGGTCTGTTAATAATATAGTAATAGCT  33
Template        597   .GA.............................  565
```

>ON849056.1 Charybdis lucifera voucher CAGL C2 cytochrome c oxidase subunit I (COX1) gene, partial cds; mitochondrial

```
product length = 139
Forward primer  1     TTAATATACGGTCATTTGGTATGAGTATAGATC  33
Template        459   .........T..C..C.....A..........  491

Reverse primer  1     AAGTTTCGGTCTGTTAATAATATAGTAATAGCT  33
Template        597   ........A.......G...............  565
```

>MZ393909.1 Charybdis lucifera isolate PTU2 cytochrome c oxidase subunit I (COX1) gene, partial cds; mitochondrial

```
product length = 139
Forward primer  1     TTAATATACGGTCATTTGGTATGAGTATAGATC  33
Template        459   .........T..C..C.....A..........  491

Reverse primer  1     AAGTTTCGGTCTGTTAATAATATAGTAATAGCT  33
Template        597   ........A.......G...............  565
```

>MW143522.1 Charybdis lucifera voucher ZSI/SRC C-180 cytochrome c oxidase subunit I (COX1) gene, partial cds; mitochondrial

```
product length = 139
Forward primer  1     TTAATATACGGTCATTTGGTATGAGTATAGATC  33
Template        459   .........T..C..C.....A..........  491

Reverse primer  1     AAGTTTCGGTCTGTTAATAATATAGTAATAGCT  33
Template        597   ........A.......G...............  565
```

>MK561421.1 Charybdis lucifera cytochrome oxidase subunit I gene, partial cds; mitochondrial

```
product length = 139
Forward primer  1     TTAATATACGGTCATTTGGTATGAGTATAGATC  33
Template        424   .........T..C..C.....A..........  456

Reverse primer  1     AAGTTTCGGTCTGTTAATAATATAGTAATAGCT  33
Template        562   ........A.......G...............  530
```

>MK530702.1 Charybdis sp. JP-2019 cytochrome oxidase subunit I gene, partial cds; mitochondrial

```
product length = 139
Forward primer  1     TTAATATACGGTCATTTGGTATGAGTATAGATC  33
Template        458   .........T..C..C.....A..........  490

Reverse primer  1     AAGTTTCGGTCTGTTAATAATATAGTAATAGCT  33
```

```
Template        596  ........A.......G................  564
```

>[MT278107.1](https://www.ncbi.nlm.nih.gov/nuccore/MT278107.1) Charybdis lucifera voucher C1803SM-10 cytochrome c oxidase subunit I (COX1) gene, partial cds; mitochondrial

```
product length = 139
Forward primer  1    TTAATATACGGTCATTTGGTATGAGTATAGATC  33
Template        392  ..........T..C..C.....A..........  424

Reverse primer  1    AAGTTTCGGTCTGTTAATAATATAGTAATAGCT  33
Template        530  ........A.......G................  498
```

>MT852943.1 Ehecatusa mixtepensis voucher CNCR 309 cytochrome c oxidase subunit I (COX1) gene, partial cds; mitochondrial

```
product length = 139
Forward primer  1    TTAATATACGGTCATTTGGTATGAGTATAGATC  33
Template        416  ..........C.....C..A..A.C........  448

Reverse primer  1    AAGTTTCGGTCTGTTAATAATATAGTAATAGCT  33
Template        554  .GA..............................  522
```

>LC537821.1 Sesarmops impressus SS63 mitochondrial COI gene for cytochrome oxidase subunit I, partial cds

```
product length = 139
Forward primer  1    TTAATATACGGTCATTTGGTATGAGTATAGATC  33
Template        459  ..........A..T.A......A.C........  491

Reverse primer  1    AAGTTTCGGTCTGTTAATAATATAGTAATAGCT  33
Template        597  ..A.............................A  565
```

>LC537819.1 Sesarmops impressus SOm4 mitochondrial COI gene for cytochrome oxidase subunit I, partial cds

```
product length = 139
Forward primer  1    TTAATATACGGTCATTTGGTATGAGTATAGATC  33
Template        459  ..........A..T.A......A.C........  491

Reverse primer  1    AAGTTTCGGTCTGTTAATAATATAGTAATAGCT  33
Template        597  ..A.............................A  565
```

>MK260144.1 Austrothelphusa sp. ww23252 cytochrome oxidase subunit I (CO1) gene, partial cds; mitochondrial

```
product length = 139
Forward primer  1    TTAATATACGGTCATTTGGTATGAGTATAGATC  33
Template        219  ..........C..T.A......A.C........  251

Reverse primer  1    AAGTTTCGGTCTGTTAATAATATAGTAATAGCT  33
Template        357  ........A.......................A  325
```

>MK260141.1 Austrothelphusa sp. ww23249 cytochrome oxidase subunit I (CO1) gene, partial cds; mitochondrial

```
product length = 139
Forward primer  1    TTAATATACGGTCATTTGGTATGAGTATAGATC  33
Template        219  ..........C..T.A......A.C........  251

Reverse primer  1    AAGTTTCGGTCTGTTAATAATATAGTAATAGCT  33
Template        357  ........A.......................A  325
```

>MK260137.1 Austrothelphusa sp. ww23240 cytochrome oxidase subunit I (CO1) gene, partial cds; mitochondrial

```
product length = 139
Forward primer  1    TTAATATACGGTCATTTGGTATGAGTATAGATC  33
Template        219  ..........C..T.A......A.C........  251

Reverse primer  1    AAGTTTCGGTCTGTTAATAATATAGTAATAGCT  33
Template        357  ........A.......................A  325
```

>KX060343.1 Charybdis hellerii isolate XDNA - 506 443 cytochrome oxidase subunit 1 (COI) gene, partial cds; mitochondrial

```
product length = 139
Forward primer  1    TTAATATACGGTCATTTGGTATGAGTATAGATC  33
Template        473  ..........C..C..C.....A........C.  505

Reverse primer  1    AAGTTTCGGTCTGTTAATAATATAGTAATAGCT  33
Template        611  ..A.............................G  579
```

>KX060333.1 Charybdis hellerii isolate XDNA - 1039 422 cytochrome oxidase subunit 1 (COI) gene, partial cds; mitochondrial

```
product length = 139
Forward primer  1    TTAATATACGGTCATTTGGTATGAGTATAGATC  33
Template        473  .......G..C..C........A........C.  505

Reverse primer  1    AAGTTTCGGTCTGTTAATAATATAGTAATAGCT  33
Template        611  ..A.............................G  579
```

>KX060327.1 Charybdis hellerii isolate XDNA - 506 370 cytochrome oxidase subunit 1 (COI) gene, partial cds; mitochondrial

```
product length = 139
Forward primer  1    TTAATATACGGTCATTTGGTATGAGTATAGATC  33
Template        473  .........C..C........A..C.....C.  505

Reverse primer  1    AAGTTTCGGTCTGTTAATAATATAGTAATAGCT  33
Template        611  ..A.............................G  579
```

>KX060320.1 Charybdis hellerii isolate XDNA - 1039 361 cytochrome oxidase subunit 1 (COI) gene, partial cds; mitochondrial

```
product length = 139
Forward primer  1    TTAATATACGGTCATTTGGTATGAGTATAGATC  33
Template        473  ......G..C..C........A........C.  505

Reverse primer  1    AAGTTTCGGTCTGTTAATAATATAGTAATAGCT  33
Template        611  ..A.............................G  579
```

>KX060300.1 Charybdis hellerii isolate XDNA - 338 279 cytochrome oxidase subunit 1 (COI) gene, partial cds; mitochondrial

```
product length = 139
Forward primer  1    TTAATATACGGTCATTTGGTATGAGTATAGATC  33
Template        443  .........C..C........A.A......C.  475

Reverse primer  1    AAGTTTCGGTCTGTTAATAATATAGTAATAGCT  33
Template        581  ..A.............................G  549
```

>KX060288.1 Charybdis hellerii isolate XDNA - 316 222 cytochrome oxidase subunit 1 (COI) gene, partial cds; mitochondrial

```
product length = 139
Forward primer  1    TTAATATACGGTCATTTGGTATGAGTATAGATC  33
Template        422  ......G..C..C........A........C.  454

Reverse primer  1    AAGTTTCGGTCTGTTAATAATATAGTAATAGCT  33
Template        560  ..A.............................G  528
```

>KX060287.1 Charybdis hellerii isolate XDNA - 275 221 cytochrome oxidase subunit 1 (COI) gene, partial cds; mitochondrial

```
product length = 139
Forward primer  1    TTAATATACGGTCATTTGGTATGAGTATAGATC  33
Template        383  ......G..C..C........A........C.  415

Reverse primer  1    AAGTTTCGGTCTGTTAATAATATAGTAATAGCT  33
Template        521  ..A.............................G  489
```

>KX060286.1 Charybdis hellerii isolate XDNA - 275 219 cytochrome oxidase subunit 1 (COI) gene, partial cds; mitochondrial

```
product length = 139
Forward primer  1    TTAATATACGGTCATTTGGTATGAGTATAGATC  33
Template        464  ......G..C..C........A........C.  496

Reverse primer  1    AAGTTTCGGTCTGTTAATAATATAGTAATAGCT  33
Template        602  ..A.............................G  570
```

>KX060274.1 Charybdis hellerii isolate XDNA - 230 186 cytochrome oxidase subunit 1 (COI) gene, partial cds; mitochondrial

```
product length = 139
Forward primer  1    TTAATATACGGTCATTTGGTATGAGTATAGATC  33
Template        467  ......G..C..C........A........C.  499

Reverse primer  1    AAGTTTCGGTCTGTTAATAATATAGTAATAGCT  33
Template        605  ..A.............................G  573
```

>KX060273.1 Charybdis hellerii isolate XDNA - 230 185 cytochrome oxidase subunit 1 (COI) gene, partial cds; mitochondrial

```
product length = 139
Forward primer  1    TTAATATACGGTCATTTGGTATGAGTATAGATC  33
Template        458  ......G..C..C........A........C.  490

Reverse primer  1    AAGTTTCGGTCTGTTAATAATATAGTAATAGCT  33
Template        596  ..A.............................G  564
```

>KX060256.1 Charybdis hellerii isolate XDNA - 233 197 cytochrome oxidase subunit 1 (COI) gene, partial cds; mitochondrial

```
product length = 139
Forward primer  1    TTAATATACGGTCATTTGGTATGAGTATAGATC  33
Template        473  ......G..C..C........A........C.  505

Reverse primer  1    AAGTTTCGGTCTGTTAATAATATAGTAATAGCT  33
Template        611  ..A.............................G  579
```

>KX060249.1 Charybdis hellerii isolate XDNA - 197 148 cytochrome oxidase subunit 1 (COI) gene, partial cds; mitochondrial

```
product length = 139
Forward primer  1    TTAATATACGGTCATTTGGTATGAGTATAGATC  33
Template        464  ......G..C..C........A........C.  496

Reverse primer  1    AAGTTTCGGTCTGTTAATAATATAGTAATAGCT  33
```

```
Template        602  ..A.............................G  570
```

>KX060244.1 Charybdis hellerii isolate XDNA - 187 130 cytochrome oxidase subunit 1 (COI) gene, partial cds; mitochondrial

```
product length = 139
Forward primer  1    TTAATATACGGTCATTTGGTATGAGTATAGATC  33
Template        473  .......G..C..C........A........C.  505

Reverse primer  1    AAGTTTCGGTCTGTTAATAATATAGTAATAGCT  33
Template        611  ..A.............................G  579
```

>KX060235.1 Charybdis hellerii isolate XDNA - 187 119 cytochrome oxidase subunit 1 (COI) gene, partial cds; mitochondrial

```
product length = 139
Forward primer  1    TTAATATACGGTCATTTGGTATGAGTATAGATC  33
Template        470  .......G..C..C........A........C.  502

Reverse primer  1    AAGTTTCGGTCTGTTAATAATATAGTAATAGCT  33
Template        608  ..A.............................G  576
```

>KX060227.1 Charybdis hellerii isolate XDNA - 172 108 cytochrome oxidase subunit 1 (COI) gene, partial cds; mitochondrial

```
product length = 139
Forward primer  1    TTAATATACGGTCATTTGGTATGAGTATAGATC  33
Template        473  .......G..C..C........A........C.  505

Reverse primer  1    AAGTTTCGGTCTGTTAATAATATAGTAATAGCT  33
Template        611  ..A.............................G  579
```

>LC547019.1 Sesarmops impressus SOm7 mitochondrial COI gene for cytochrome oxidase subunit I, partial cds

```
product length = 139
Forward primer  1    TTAATATACGGTCATTTGGTATGAGTATAGATC  33
Template        459  ..........A..T.A......A.C........  491

Reverse primer  1    AAGTTTCGGTCTGTTAATAATATAGTAATAGCT  33
Template        597  ..A.............................A  565
```

>OM714500.1 Monomia argentata voucher MNHN-IU-2014-10076 cytochrome c oxidase subunit I (COX1) gene, partial cds; mitochondrial

```
product length = 139
Forward primer  1    TTAATATACGGTCATTTGGTATGAGTATAGATC  33
Template        413  ..........T..C........A..G.....C.  445

Reverse primer  1    AAGTTTCGGTCTGTTAATAATATAGTAATAGCT  33
Template        551  ..A.................C............  519
```

>OK465141.1 Aedes vexans voucher IZBE0210183 cytochrome c oxidase subunit I (COX1) gene, partial cds; mitochondrial

```
product length = 139
Forward primer  1    TTAATATACGGTCATTTGGTATGAGTATAGATC  33
Template        459  ..........A....C......T.C.T......  491

Reverse primer  1    AAGTTTCGGTCTGTTAATAATATAGTAATAGCT  33
Template        597  ..A........A.....................  565
```

>OK465140.1 Aedes vexans voucher IZBE0210182 cytochrome c oxidase subunit I (COX1) gene, partial cds; mitochondrial

```
product length = 139
Forward primer  1    TTAATATACGGTCATTTGGTATGAGTATAGATC  33
Template        459  ..........A....C......T.C.T......  491

Reverse primer  1    AAGTTTCGGTCTGTTAATAATATAGTAATAGCT  33
Template        597  ..A........A.....................  565
```

>OK165449.1 Tehuana lamothei voucher CNCR 8812 cytochrome c oxidase subunit I (COX1) gene, partial cds; mitochondrial

```
product length = 139
Forward primer  1    TTAATATACGGTCATTTGGTATGAGTATAGATC  33
Template        416  ..........T........A..A.C......C.  448

Reverse primer  1    AAGTTTCGGTCTGTTAATAATATAGTAATAGCT  33
Template        554  .G.....................G.........  522
```

>MT590670.1 Ocypode macrocera voucher Cr20 cytochrome c oxidase subunit I (COX1) gene, partial cds; mitochondrial

```
product length = 139
Forward primer  1    TTAATATACGGTCATTTGGTATGAGTATAGATC  33
Template        454  ..........A..C........A.C......C.  486

Reverse primer  1    AAGTTTCGGTCTGTTAATAATATAGTAATAGCT  33
Template        592  .G.........A.....................  560
```

>MZ393879.1 Monomia argentata isolate MON4 cytochrome c oxidase subunit I (COX1) gene, partial cds; mitochondrial

```
product length = 139
Forward primer  1    TTAATATACGGTCATTTGGTATGAGTATAGATC  33
Template        459  ..........T..C........A..G.....C.  491

Reverse primer  1    AAGTTTCGGTCTGTTAATAATATAGTAATAGCT  33
Template        597  ..A.................C............  565
```

>MT075548.1 Aedes vexans isolate AT.p01.F06 cytochrome c oxidase subunit I (COX1) gene, partial cds; mitochondrial

```
product length = 139
Forward primer  1    TTAATATACGGTCATTTGGTATGAGTATAGATC  33
Template        418  ..........A....C......T.C.T......  450

Reverse primer  1    AAGTTTCGGTCTGTTAATAATATAGTAATAGCT  33
Template        556  ..A........A.....................  524
```

>MT852050.1 Villalobosius lopezformenti voucher CNCR 10034 cytochrome c oxidase subunit I (COX1) gene, partial cds; mitochondrial

```
product length = 139
Forward primer  1    TTAATATACGGTCATTTGGTATGAGTATAGATC  33
Template        416  .........AT........A..A.C........  448

Reverse primer  1    AAGTTTCGGTCTGTTAATAATATAGTAATAGCT  33
Template        554  ..A.....A........................  522
```

>MT852942.1 Pseudothelphusa pecki voucher CNCR 16776 cytochrome c oxidase subunit I (COX1) gene, partial cds; mitochondrial

```
product length = 139
Forward primer  1    TTAATATACGGTCATTTGGTATGAGTATAGATC  33
Template        416  ..........C........A..A.C......C.  448

Reverse primer  1    AAGTTTCGGTCTGTTAATAATATAGTAATAGCT  33
Template        554  .G.....................G.........  522
```

>MT001388.1 Aedes vexans isolate AT.p03.B03 cytochrome c oxidase subunit I (COX1) gene, partial cds; mitochondrial

```
product length = 139
Forward primer  1    TTAATATACGGTCATTTGGTATGAGTATAGATC  33
Template        418  ..........A....C......T.C.T......  450

Reverse primer  1    AAGTTTCGGTCTGTTAATAATATAGTAATAGCT  33
Template        556  ..A........A.....................  524
```

>MT001303.1 Aedes vexans isolate AT.p01.B06 cytochrome c oxidase subunit I (COX1) gene, partial cds; mitochondrial

```
product length = 139
Forward primer  1    TTAATATACGGTCATTTGGTATGAGTATAGATC  33
Template        418  ..........A....C......T.C.T......  450

Reverse primer  1    AAGTTTCGGTCTGTTAATAATATAGTAATAGCT  33
Template        556  ..A........A.....................  524
```

>MN184690.1 Charybdis feriata isolate Biosec737 cytochrome oxidase subunit I (COI) gene, partial cds; mitochondrial

```
product length = 139
Forward primer  1    TTAATATACGGTCATTTGGTATGAGTATAGATC  33
Template        459  ....C.....C..T........A..A.......  491

Reverse primer  1    AAGTTTCGGTCTGTTAATAATATAGTAATAGCT  33
Template        597  ..A..A...........................  565
```

>MN407152.1 Phoridae sp. isolate UGC0011557 cytochrome oxidase subunit I (cox1) gene, partial cds; mitochondrial

```
product length = 139
Forward primer  1    TTAATATACGGTCATTTGGTATGAGTATAGATC  33
Template        458  ...............C......T.C.T.T....  490

Reverse primer  1    AAGTTTCGGTCTGTTAATAATATAGTAATAGCT  33
Template        596  ..A.....A........................  564
```

>NC_040977.1 Nanosesarma minutum voucher NM mitochondrion, complete genome

```
product length = 139
Forward primer  1    TTAATATACGGTCATTTGGTATGAGTATAGATC  33
Template        500  .C.............A......A.C......C.  532

Reverse primer  1    AAGTTTCGGTCTGTTAATAATATAGTAATAGCT  33
Template        638  ..A....................T.........  606
```

Products on target templates

If you want to allow any of the unintended targets, check the box(es) next to the ones you accept and try again to re-search for specific primers

Submit

Help

FOLLOW NCBI

Connect with NLM

National Library of Medicine
8600 Rockville Pike
Bethesda, MD 20894

Web Policies
FOIA
HHS Vulnerability Disclosure

Help
Accessibility
Careers

NLM NIH HHS USA.gov
